# Active Degradation Explains the Distribution of Nuclear Proteins during Cellular Senescence

**DOI:** 10.1371/journal.pone.0118442

**Published:** 2015-06-26

**Authors:** Enrico Giampieri, Marco De Cecco, Daniel Remondini, John Sedivy, Gastone Castellani

**Affiliations:** 1 Department of Physics and Astronomy, Bologna University, Bologna, Italy and INFN Bologna; 2 Department of Molecular Biology, Cell Biology and Biochemistry, Center for Genomics and Proteomics, Brown University, Providence, RI, USA; Novartis Vaccines, UNITED STATES

## Abstract

The amount of cellular proteins is a crucial parameter that is known to vary between cells as a function of the replicative passages, and can be important during physiological aging. The process of protein degradation is known to be performed by a series of enzymatic reactions, ranging from an initial step of protein ubiquitination to their final fragmentation by the proteasome. In this paper we propose a stochastic dynamical model of nuclear proteins concentration resulting from a balance between a constant production of proteins and their degradation by a cooperative enzymatic reaction. The predictions of this model are compared with experimental data obtained by fluorescence measurements of the amount of nuclear proteins in murine tail fibroblast (MTF) undergoing cellular senescence. Our model provides a three-parameter stationary distribution that is in good agreement with the experimental data even during the transition to the senescent state, where the nuclear protein concentration changes abruptly. The estimation of three parameters (cooperativity, saturation threshold, and maximal velocity of the reaction), and their evolution during replicative passages shows that only the maximal velocity varies significantly. Based on our modeling we speculate the reduction of functionality of the protein degradation mechanism as a possible competitive inhibition of the proteasome.

## Introduction

Modeling changes in cellular states, such as differentiation, is one of the primary goals of systems biology. A large amount of effort is spent to model such processes in ways that are complete enough to be useful while being simple enough to be understood. Efforts of this kind have to balance the accuracy of the modeling of each single process with the complexity, both mathematical and algorithmical, of bringing these models together. One common practice is to employ a simple description of each process in order to subsequently combine them into a single macro-model.

In the last years much interest has centered on borrowing stochastic techniques from other fields and applying them in systems biology. It became clear that the biochemical fluctuations of individual reactions in the cell are not a secondary effect, but rather a driving force that the cell has to circumvent, or sometimes exploit, to survive.

Simple stochastic models that can be easily understood and verified with *ad hoc* experiments thus represent valuable approaches to describe the mesoscopic behavior of life processes. The problem under study is the amount of nuclear proteins as a function of replicative passages. This quantity is known to vary with cellular senescence [1–3], and reflects the overall functionality of the cell [4, 5]. The steady state quantity of proteins is the consequence of the balance of two opposing processes: protein synthesis and protein degradation. The accumulation of oxidized, misfolded, ubiquitinated and aggregated proteins during cellular senescence is well documented [6–8] and is believed to be detrimental for cell viability. This process is hard to properly measure due to experimental difficulties [9], and we may have to resort to indirect measurements to evaluate its functionality.

We develop here a model that describes protein degradation as an active process, in agreement with the large literature on this subject [10]. With the term “active process” we refer to a birth-death process of the proteins in the nucleus, where the death rate is not linear in the number of proteins. A death rate linear in the number of proteins would indicate a passive degradation, where each protein deteriorates independently from the others. This is clearly a non realistic hypothesis; A closer approximation of this process (retaining the simplicity) can be an enzymatic kinetic rate, that implies an external process of removal that is not independent from the protein number or concentration. In the simplest case, a Michaelis-Menten kinetics, this leads to a negative binomial distribution of total protein amount, similar to that used to model the amount of a single protein. This can be easily generalized to include the presence of cooperativity in the enzymatic reactions of the degradation process [8].

We generated a large volume of high resolution fluorescence microscopy data, based on single-cell image analysis. These experiments followed the replicative senescence of murine tail fibroblast (MTF) until the complete senescence ensued. We performed a fluorescent staining on the nuclear proteins of the cells, that are known to vary with the cellular senescence and can be characterized from an experimental point of view with a more robust procedure. These observations have been used to validate the ability of the model to describe the protein distribution and to evaluate how this distribution changes when cells approach senescence.

In the next section we will discuss the experiment performed and the model, showing the exact resolution for the simplest case, and defining how to obtain a numerical solution for the general case. We will also show how the model is capable of reproducing experimental data. We compare a null model obtained from the production of a single protein with our model, proving that the latter obtains a better performance. Using the estimated parameters we will provide some insights into the cellular senescence process as a reduction of the efficiency of protein degradation, which can be interpreted in the framework of enzyme inhibition.

There is, indeed, considerable agreement on the substantial age-associated accumulation of nuclear protein in cultured cells [11–13]. Our model successfully accounts for a decrease in protein degradation, and explains the data using a cooperative mechanism.

## Results

### Nuclear protein content of aging mouse fibroblast in culture

We first corroborated previous works that senescent cells contain more nuclear protein using mouse tail fibroblasts (MTF), passaged as previously reported ([11]). We quantified nuclear protein content with single cell fluorescence microscopy, and subsequently through cellular fractioning followed by protein isolation from nuclear extract. These data are in large agreement with our previous findings.

In order to study the kinetic of the accumulation of nuclear protein in the nucleus without biases, we used single-cell quantitative microscopy for every cellular passage, corresponding to two population doublings in our passaging regime (see Methods). In our hands MTFs consistently reached senescence after 12–13 passages. Nuclear protein content was quantified per each passage (S1 Table): it resulted in a progressive increase positively correlated with cellular age.

### Burst production model

Even if the protein production process is a rather complex one, recent experimental [14–16] and theoretical considerations suggests that it may be approximated with a simple model, by focusing just on the crucial steps of mRNA and protein production. In this model protein turnover is represented by the following reaction scheme:

$$\mathrm{DNA}\overset{k_{1}}{\to }\underset{\underset{\emptyset }{↓}\gamma_{1}}{\mathrm{mRNA}}\overset{k_{2}}{\to }\underset{\underset{\emptyset }{↓}\gamma_{2}}{\mathrm{Protein}}$$

Where the DNA quantity is assumed constant and mRNA production is low. The reaction constants *k*_1_ and *k*_2_ represent the production rates of mRNA and protein respectively, and the *γ*_1_ and *γ*_2_ their degradation rates. For each mRNA molecule, several proteins are produced, generating the so-called protein production burst. These bursts have been experimentally observed [16] and show an exponential distribution, as expected from the model above in the limit of short-lived mRNA (*γ*_1_ greater than *k*_1_).

The above model has been solved in the continuous limit by Friedman et al [17], which started from a generic mono-dimensional Fokker-Planck equation for the concentration of protein *p*(*x*) with *x* = *n*/*V*, where *V* is the volume of the cell and *n* the protein number.

They have shown that under the hypothesis of independent exponential bursts of RNA, this model has a stationary distribution described by a Gamma distribution

$$p\left(x\right)=\frac{x^{a-1}e^{-x/b}}{b^{a}\Gamma \left(a\right)}$$

where the parameter *a* = *k*_1_/*γ*_2_ represents the average number of production bursts per cycle and *b* = *k*_2_/*γ*_1_ is the average number of protein produced by each burst.

The Gamma distribution is commonly used to describe over-disperse distributions, with a Fano factor (ratio between the variance and the mean of the distribution) greater than 1. A Fano factor of 1 is characteristic of a very simple process of independent creation and destruction of a protein. For a single enzyme *Friedman et al*. [17] showed a good agreement with experimental data. One could argue that being the parameter *b* similar for all the proteins, the distribution of the total protein would simply be given by the sum of the distribution of each protein, as:

$$\sum_{i}\Gamma \left(a_{i},b\right)=\Gamma \left(\sum_{i}a_{i},b\right).$$

We will use this as the null model to be compared with ours.

In these models the protein levels that are predicted are all the proteins that are produced and not completely destroyed, including the ubiquinated ones. This is compatible to the experimental setting used in this work, that measure the signal from all the protein in the nucleus, without distinction between the ubiquination state.

This crude approximation of the protein production rate can be justified considering that is known that there is little correlation between the transcript levels and the corresponding protein abundance [18–20]. It is also known that the protein production is almost an order of magnitude faster than the protein degradation [21], allowing this process to the replaced with its average value instead of at the instantaneous value. Recent measures [22] also suggest that the individual proteins are subjects of a negative feedback loop that reduce the protein production noise at the level of single proteins, enhancing the stability of the whole proteome production. In the Results section it will be shown that this distribution cannot describe the actual distribution of proteins, which is much more peaked and asymmetric.

### Active degradation model

Our model will be described and investigated in the framework of the Chemical Master Equation [23, 24]. The Chemical Master Equation is a stochastic modeling approach that describes the model by the probability of occupancy of each of its available states. This means that the evolution of a non-linear, discrete, stochastic system is divided into a huge number (in general infinity) of linear, deterministic ordinary differential equations. This modeling approach is especially suited to describing biochemical processes inside the cell [25–34], that are known to be driven by small numbers of discrete entities, like genes [35, 36], RNA transcript [37] and proteins [38, 39]. This has been proven to be useful in describing non-trivial stochastic effects on the classical dynamics that describe biochemical processes, with effects like stochastic resonance, stochastic focusing and so on [29, 40–45]. The role of this approach is becoming more relevant since we are now able to observe low level details of the internal behavior of the cell, from the individual cell genetic expression [14–16, 28] down to the individual RNA molecule [46].

The master equation model describes discrete valued processes, so we will refer not to the Gamma distribution, but to its discrete equivalent, the Negative Binomial distribution. This change in the model does not change the validity of the results. The relationship between the two has been addressed by Paulsson et al [47], but can be simplified as follows: as the Gamma distribution can be seen as the sum of independent Exponential distributions, the Negative Binomial can be seen as the sum of independent Geometrical distributions, the discrete equivalent of the Exponential distribution.

We aim to describe the amount of proteins in the cell nucleus as a coarse-grained process of generation and degradation, without differentiation between individual protein species. Considering the total production of proteins as the sum of many weakly correlated processes, the total effect can be seen as a quasi-stationary process with a mean value greater than its standard deviation, so we will approximate it as a constant production.

The degradation process, on the other hand, is driven by a much smaller number of reactions, each of which is strongly correlated with the others: the target protein is first ubiquitinated, then moved to a different location and finally degraded by the proteasome (a large degradation complex that binds to the target protein and fragments it). As a first approximation we will consider all these processes as an enzymatic process performed in a single step.

This hypothesis is based on the observation that in mammalian cells protein degradation is an active process. In this model we ignore the effect of the dilution due to cellular division, being the process time scale much faster than the cell division time, as several weeks can be spent between two divisions in the late stages of cellular senescence.

We can express this process with a monodimensional master equation in the form:

$$\partial_{t}P_{n}\left(t\right)=\left(E_{n}^{-}-1\right)g_{n}P_{n}\left(t\right)+\left(E_{n}^{+}-1\right)r_{n}P_{n}\left(t\right)$$ (1)

that expresses the temporal evolution of the probability *P*_*n*_ of observing *n* proteins in the nucleus.

The operators ${𝔼}_{n}^{\pm }$ are the unitary step operator defined as ${𝔼}_{n}^{+}f(n)=f(n+1)$ and ${𝔼}_{n}^{-}f(n)=f(n-1)$ where *f* is an integer valued function.

The *g*_*n*_ and *r*_*n*_ are the generation and recombination [23] transition rates:

$$\begin{array}{ccc} g_{n} & = & \beta \\ r_{n} & = & \gamma^{\prime }\frac{n}{\theta +n}. \end{array}$$ (2)

The *g*_*n*_ represents the constant production rate due to the translation of the RNA into proteins, while the *r*_*n*_ represents the active degradation of the protein by means of the degradation mechanisms.

This master equation reaches a stationary distribution under the convergence condition that *γ*′ > *β*, i.e. the rate of maximum degradation is higher than the constant production rate.

For all practical purposes we can normalize all the kinetic coefficients to the value of *β*, by defining $\gamma =\frac{\beta }{\gamma^{\prime }}$, as we are interested only in the stationary distribution and not on the time-dependent solution:

$$\begin{array}{ccc} g_{n} & = & \gamma \\ r_{n} & = & \frac{n}{\theta +n}. \end{array}$$ (3)

We can find the stationary solution by a recurrence relation, which states that in mono-dimensional systems with one-step processes the solution is subject to the detailed balance condition:

$$P_{n}r_{n}=P_{n-1}g_{n-1}.$$ (4)

The obtained equation for the occupancy probability of each state of the system is:

$$P_{n}=P_{0}\prod_{i=1}^{n}\frac{g_{i-1}}{r_{i}}=P_{0}\prod_{i=1}^{n}\frac{\theta +i}{i}\gamma$$

where *P*(*n*) is the probability of observing *n* protein in the nucleus.

By expanding the product we can factor the formula in terms of exponentials and factorials of *n*:

Pn=P0(θ+n)!n!θ!γn=C0(θ+nn)γn (5)

where *C*_0_ is a normalization constant and the formula can be recognized as a Negative Binomial Distribution where all the terms that don’t depend on *n* have been included into *C*_0_. The use of the Negative Binomial Distribution as basic model for cellular processes has been proposed in the past on the basis of stochastic properties of the biochemical regulatory circuits [48].

Summing from *n* = 0 to infinity it yields (see appendix):

$$C_{0}={\left(1-\gamma \right)}^{\theta +1}$$

For the distribution to exist the *γ* value should be between 0 and 1, meaning that the creation rate should be less than the maximum possible degradation rate. While *θ* can assume any positive value.

The resulting distribution is monomodal and its mode can be evaluated with very good accuracy by the solution of the deterministic system where the increase and decrease terms balance out.

g(x)=r(x),x∈R

from which we can obtain the following relationship for the mode:

$$mode=\theta {\left(\frac{\gamma -1}{\gamma }\right)}^{-1}$$

### Cooperative model of active degradation

The protein degradation chain is a complex mechanism composed by several steps performed by specific cellular machinery that need to be performed in a specific order. Given that the amount of proteins responsible for the degradation chain are very diluted in respect to their target, the whole proteome, it is not far fetched to hypothesize a pseudo-stationary dynamic like the one underlying the single protein dynamic.

We use the Hill kinetics [40, 49, 50], where the degradation rate depends on the presence of a cooperative effect between degrader proteins and their protein target.

This hypothesis leads to a change in the degradation term as follow:

$$\begin{array}{ccc} g_{n} & = & \gamma \\ r_{n} & = & \frac{n^{\alpha }}{\theta^{\alpha }+n^{\alpha }}. \end{array}$$ (6)

where the *α* exponent quantifies the cooperativity effects between subunits and can be any positive real number.

$$P_{n}=P_{0}\prod_{i=1}^{n}\frac{g_{i-1}}{r_{i}}=P_{0}\prod_{i=1}^{n}\frac{\theta^{\alpha }+i^{\alpha }}{i^{\alpha }}\gamma$$ (7)

To obtain a partial solution it is necessary to decompose the term *θ* ^*α*^ + *i* ^*α*^, and this is possible only for integers valued *α*. In these case we can obtain the decomposition in terms of the solution *S*_*r*_:

$$\theta^{\alpha }+i^{\alpha }=\prod_{r=1}^{\alpha }\left(1-S_{r}\right).$$

This allows us to obtain the following form for the stationary solution:

Pn=C0γn∏r=1α(n+Srn)

where the *C*_0_ term has the form of a generalized HyperGeometric function:

$$C_{0}{=}_{\alpha }F_{\alpha -1}\left(S_{1},\ldots ,S_{\alpha },1,\ldots ,1,\gamma \right)$$.

The resulting distribution is still monomodal but depending on the value of the Hill cooperation parameter *α* it can exhibit long, heavy tails. Due to the impossibility to write a closed form for continuous–valued *α*, we will use a numerical estimation of the distribution in this work.

The constraints of *γ* and *θ* are the same as in the previous case, *γ* ∈ (0,1) and *θ* > 0. The *α* parameter can be any positive real value.

We will refer to this distribution as the generalized Negative Binomial distribution.

### Goodness of fit of the models

Using a bootstrap method we evaluate the goodness of fit of the two models of active degradation (as described in the Material and Methods section). This procedure allows us to test the hypothesis that a distribution describes the data, without biases due to the fit procedure [51]. This method has been used instead of the Kolmogorov-Smirnov test because the K-S test has a biased p-value when the tested distribution parameters have been estimated from the data.

The two distributions to be tested are the Negative Binomial distribution and the Generalized Negative Binomial, that allows cooperativity. The results are shown in Table 1 where each column represents a different passage of the cell culture (one passage being roughly equivalent to two population doublings of the culture). The first four rows represent the results for each biological replica of the experiment, while the last row, designated “combined experiments”, shows the results on the dataset obtained by the union of the data from each experiment.

**Table 1:** *p values* for the fit between the described distributions and the data. Each row represent a different experiment, that was evaluated individually. The row labeled as *combined* *experiments* is the result of the fit using all the values from all experiments for each passage (where available) to increase the size of the sample. The p value represent the hypothesys that the data comes from the distribution, so low p values indicate poor agreement. For most cases the negative binomial fit poorly the data (low p values) while the generalized version perform correctly in all cases (all p values are above 0.05).

STANDARD NEGATIVE BINOMIAL:						
	**3**	**9**	**10**	**11**	**12**	**13**
**experiment 1**	0.34					*0.02*
**experiment 2**	*0.00*					0.33
**experiment 3**	*0.00*					0.64
**experiment 4**		*0.00*	*0.01*	0.17	*0.05*	*0.01*
**combined experiments**	*0.00*	*0.00*	*0.01*	0.17	*0.05*	*0.00*
GENERALIZED NEGATIVE BINOMIAL:						
	**3**	**9**	**10**	**11**	**12**	**13**
**experiment 1**	0.17					0.07
**experiment 2**	0.22					0.21
**experiment 3**	0.85					0.11
**experiment 4**		0.25	0.55	0.3	0.57	0.18
**combined experiments**	0.44	0.25	0.55	0.3	0.57	0.16

**Fig 1:**
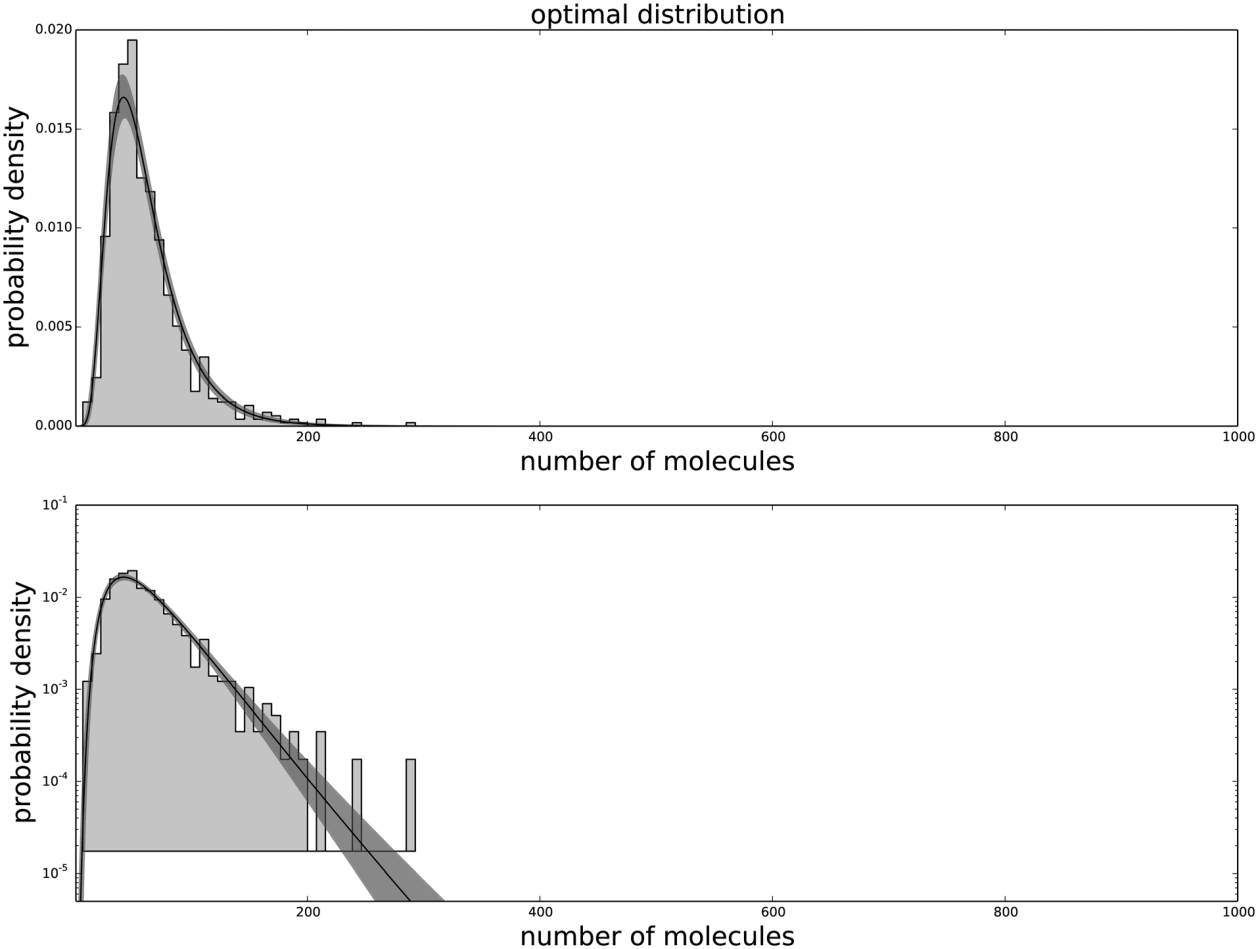
The agreement between the data and the fitted distribution for the third passage. The upper graph is linearly scaled, the lower one is logarithmically scaled to show the distribution at high n. The black line is the best estimated distribution, while the gray area represents the uncertainty in the distribution.

**Fig 2:**
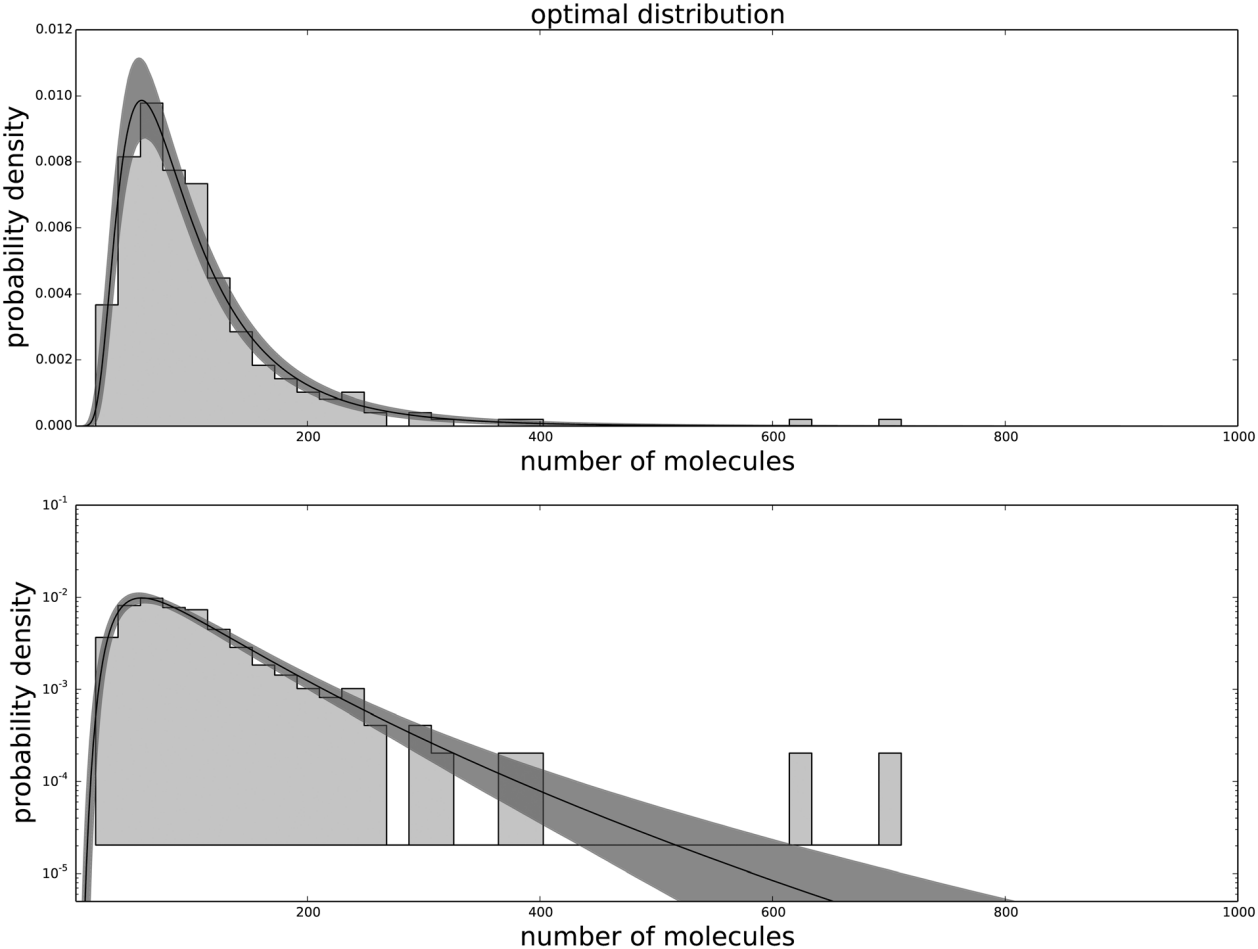
The agreement between the data and the fitted distribution for the ninth passage. The upper graph is linearly scaled, the lower one is logarithmically scaled to show the distribution at high n. The black line is the best estimated distribution, while the gray area represents the uncertainty in the distribution.

**Fig 3:**
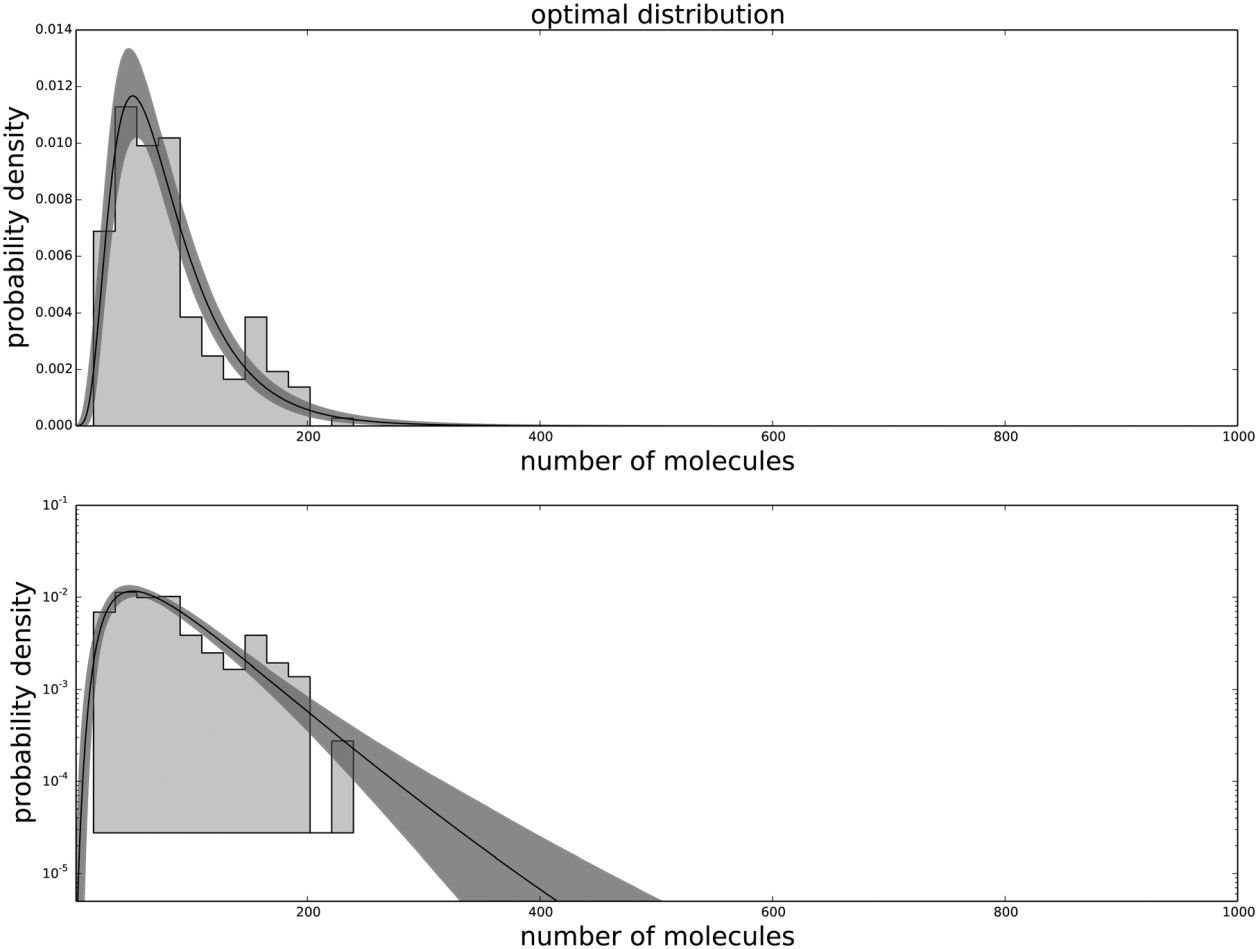
The agreement between the data and the fitted distribution for the tenth passage. The upper graph is linearly scaled, the lower one is logarithmically scaled to show the distribution at high n. The black line is the best estimated distribution, while the gray area represents the uncertainty in the distribution.

**Fig 4:**
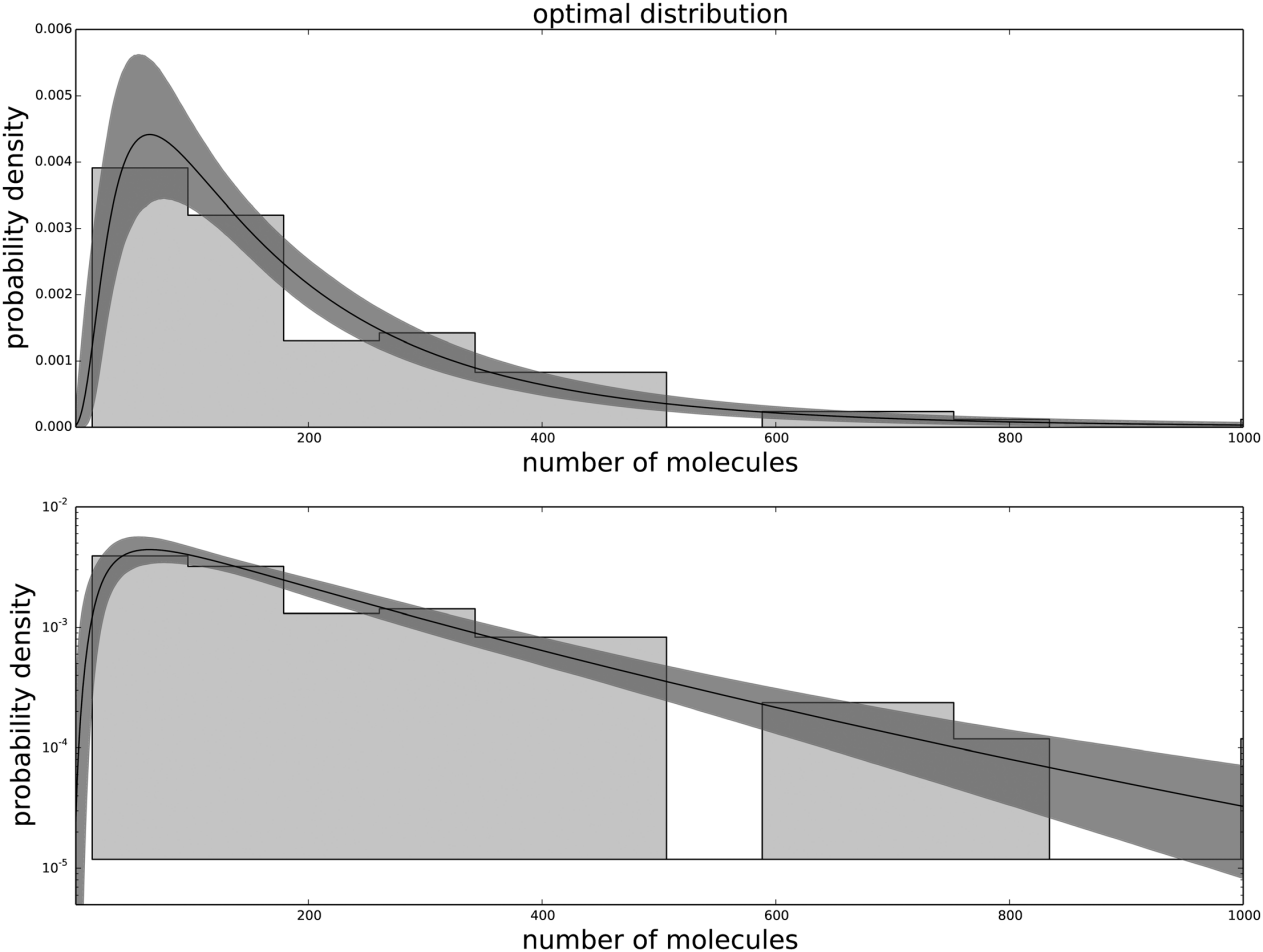
The agreement between the data and the fitted distribution for the eleventh passage. The upper graph is linearly scaled, the lower one is logarithmically scaled to show the distribution at high n. The black line is the best estimated distribution, while the gray area represents the uncertainty in the distribution.

**Fig 5:**
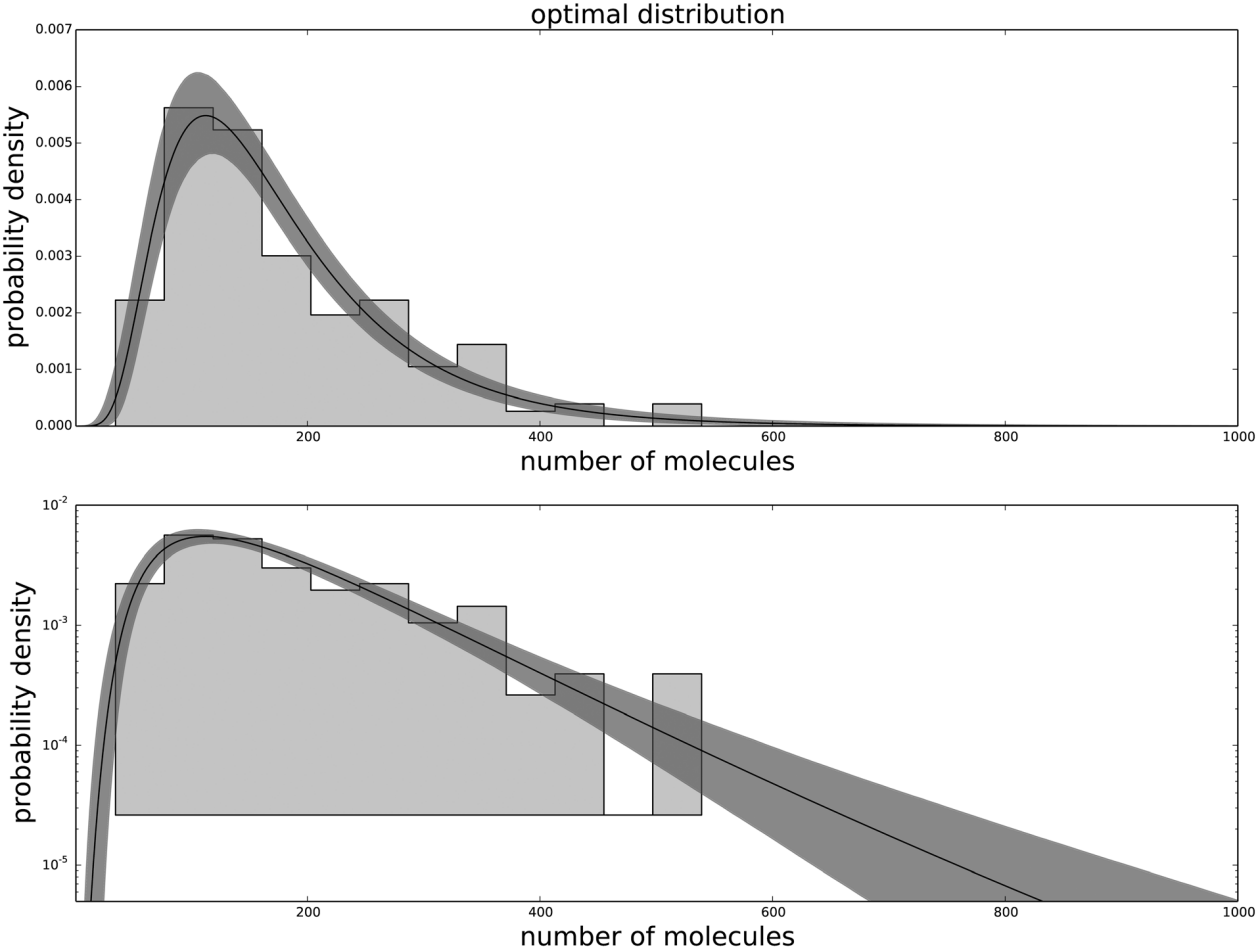
The agreement between the data and the fitted distribution for the twelfth passage. The upper graph is linearly scaled, the lower one is logarithmically scaled to show the distribution at high n. The black line is the best estimated distribution, while the gray area represents the uncertainty in the distribution.

**Fig 6:**
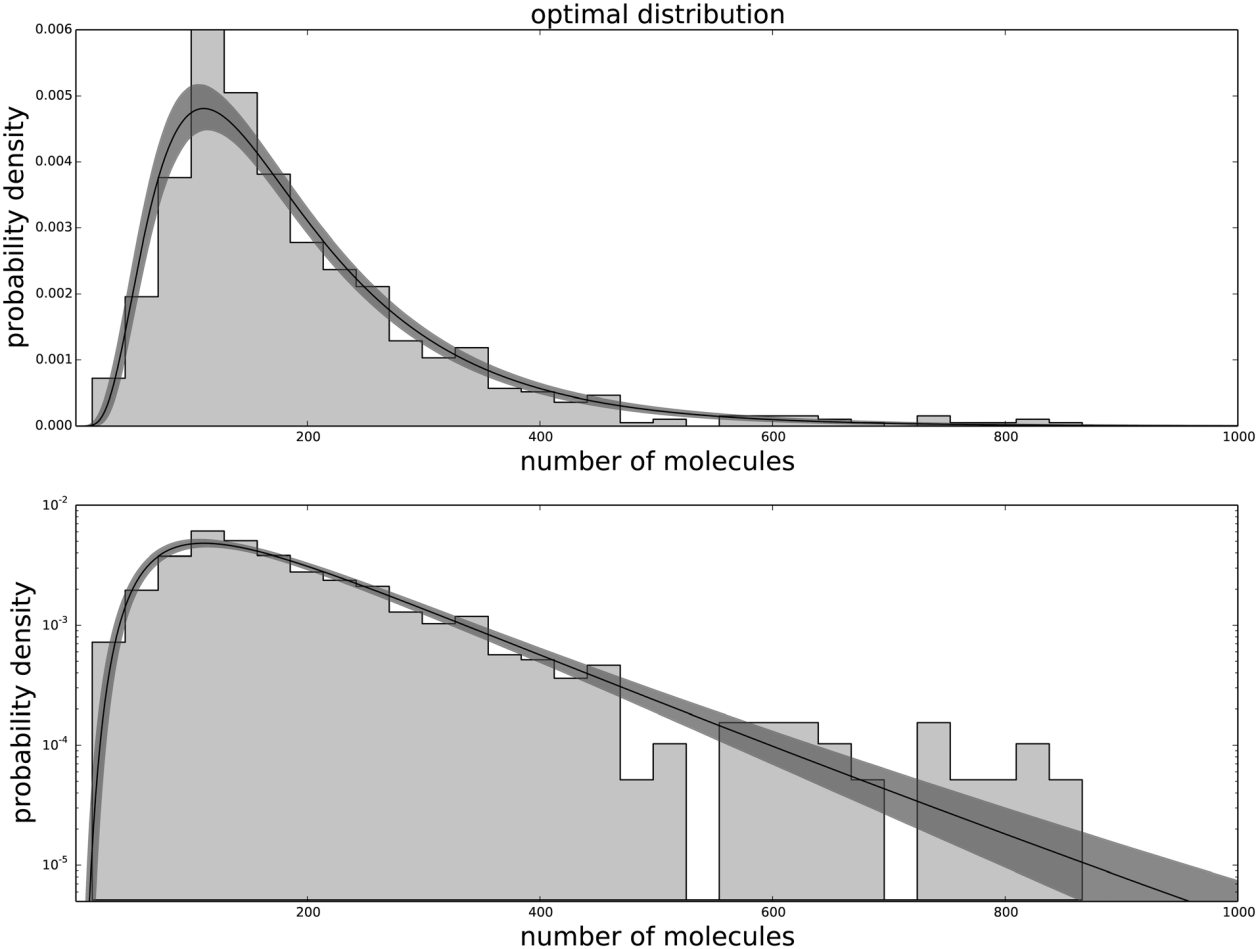
The agreement between the data and the fitted distribution for the thirthinth passage. The upper graph is linearly scaled, the lower one is logarithmically scaled to show the distribution at high n. The black line is the best estimated distribution, while the gray area represents the uncertainty in the distribution.

The Generalized Negative Binomial distribution is compatible with the observed data at all times for all the available data points (p-value > 0.05), while the standard Negative Binomial distribution does not satisfy these criteria (see Table 1). The Gamma distribution (not shown) produces results similar to the Negative Binomial distribution, hence is not in good agreement with the data. The agreement between the data and the model for each passage are shown in Fig 1 for the agreement with the third passage data, Fig 2 for the agreement with the ninth passage data, Fig 3 for the agreement with the tenth passage data, Fig 4 for the agreement with the eleventh passage data, Fig 5 for the agreement with the twelfth passage data, Fig 6 for the agreement with the thirthinth passage data.

To account for the different number of parameters in the two model distributions we performed the AIC (Aikake Information Criterion) and BIC (Bayesian Information Criterion) tests. The results in Table 2 show that the Generalized Negative Binomial distribution outperforms the negative binomial in all passages.

**Table 2:** The AIC and BIC differences between the two models. A negative value imply a preference toward the generalized negative binomial. The generalized negative binomial is preferred in all cases for both measurements, aside for the BIC value of the tenth passage where the difference in close to 0 (so they are equivalent). Bigger dataset (passages 3 and 13) evidence a strong preference toward the generalized negative binomial. These results are robust under correction for small sample size (that gives a correction of order 10^−1^). As BIC penalizes strongly the higher number of parameters of the generalized negative binomial we obtain values lower than those of the AIC, but with the same general trend.

passage	ΔAIC	ΔBIC	dataset size
03	-21.31	-16.70	744
09	-24.00	-20.46	255
10	-3.24	0.03	195
11	-3.70	-1.06	103
12	-4.36	-1.16	182
13	-25.69	-21.16	684

From now on the analysis will refer only to the Generalized Negative Binomial distribution.

### Behavior of the model parameters

**Fig 7:**
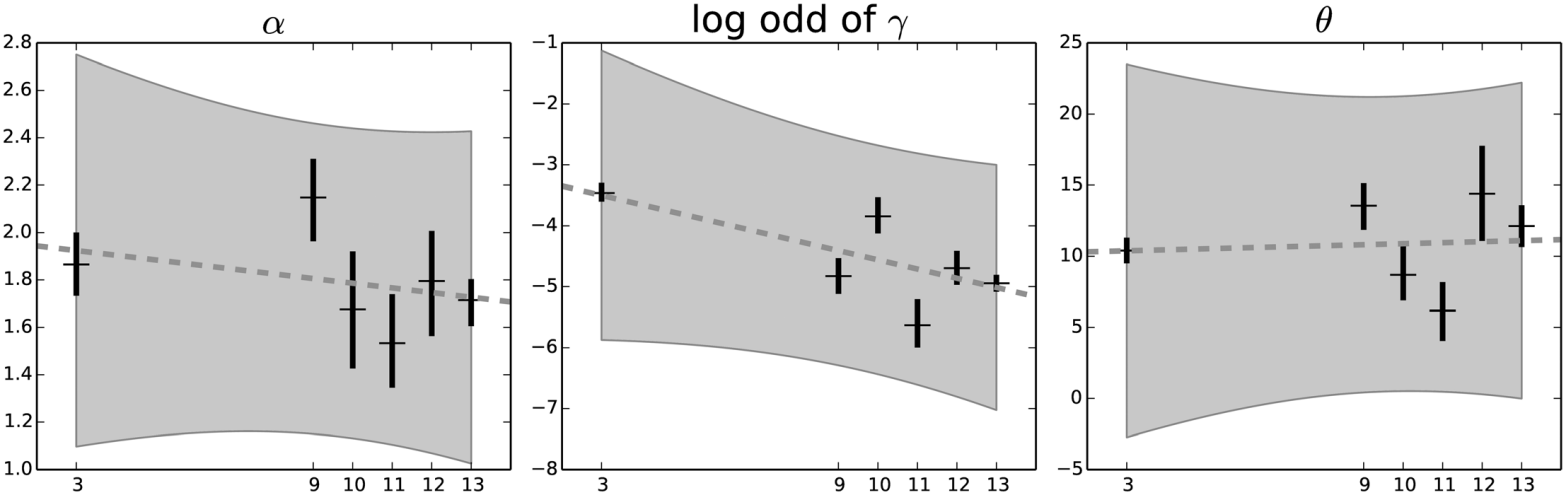
The behavior of the parameters over time. We can see that the parameters *α* and *θ* are compatible with a constant value, while the parameter *γ*, here shown as the log odd of the value, is compatible with a decrease during replicative senescence. The p value of the hypothesis of no variation during passages is 0.443 for *α*, 0.832 for *θ* and 0.048 for *γ*, evaluated with a weighted least square regression. The only parameter to varies significantly is thus *γ*, the balance between the protein creation and destruction rate.

In Fig 7 we report the estimates of the distribution parameters as a function of cell replicative passages.

The Hill threshold concentration *θ* does not change significantly through the replicative passages (p = 0.832). The Hill cooperativity coefficient *α* has a value close to 2, thus the reaction is clearly cooperative and this justifies the choice to include this parameter in our model. Moreover, its value doesn’t change significantly (p = 0.443), thus the reaction structure remains the same during senescence. To characterize the trend of the *γ* parameter, that describes the balance between the creation and the degradation maximum velocities, we analyzed the log odd ($log\frac{1-\gamma }{\gamma }$), in order to linearize and symmetrize its range. The slope of the log odd of *γ* varies significantly (p = 0.048) decreasing with the replicative passages, thus *γ* is increasing over time.

These observations are compatible with the hypothesis that the degradation chain is qualitatively the same during cellular senescence, and does not undergo structural changes, thus the protein accumulation in the nucleus is due to the variation of balance between protein creation and degradation.

**Fig 8:**
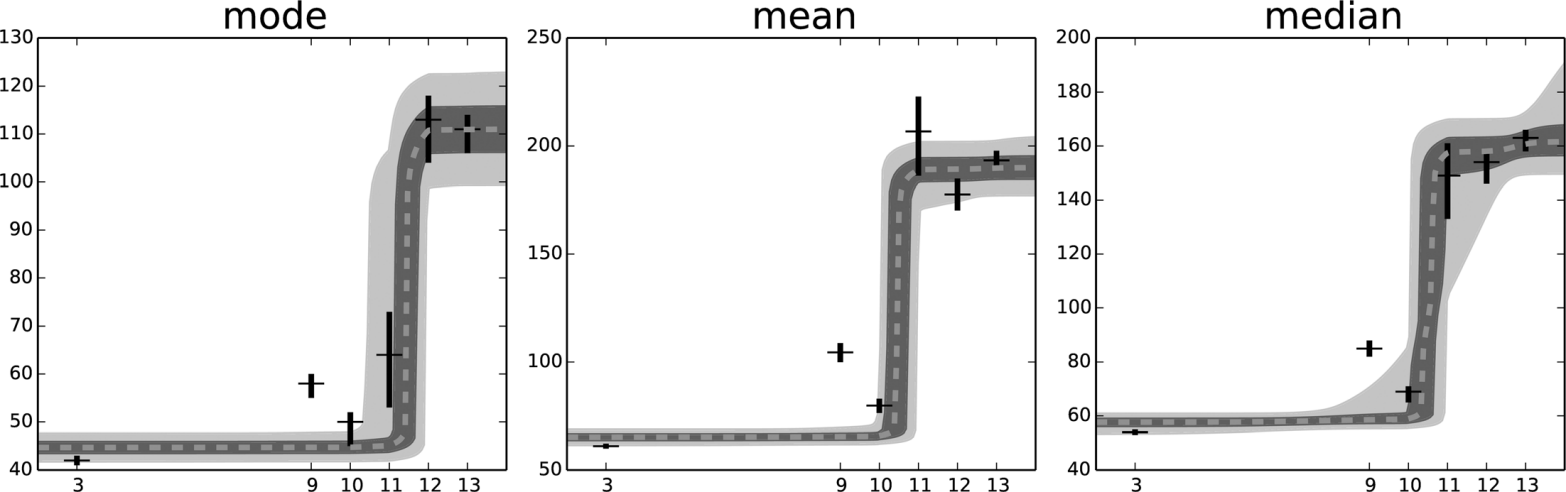
The progression in time of three different centrality measures of the fitted distribution: mode, mean and median. All of them exhibit a transition in the observed value around the eleventh passage. The gray ares are the 50% and 95% confidence interval for the fit with a logistic function with four parameters: maximum and minimum value, steepness and transition point.

In Fig 8 we report the estimation of the main centrality measures of the distribution: mean, mode and median. These centrality measures have a non–linear trend (sigmoidal), as a consequence of the changes of the distribution parameters *α*, *θ* and *γ*.

These results are compatible with an increase in the amount of nuclear proteins during cellular senescence. It is important to note that this process is not continuous, but rather a steep one, even if the underlying parameters vary in a smooth, almost linear manner. The cells undergo a transition from a state of high efficiency of protein degradation to a lower one in few replicative steps. The most relevant change occurs between the eleventh and twelfth passages, in a way that is compatible with biological markers for the onset of cellular senescence, like the fraction of SA-*β*-gal positive cells, which reaches 100% in the same passages (data not shown).

## Discussion

We proposed a model describing the amount of nuclear proteins as a production/degradation process, in which the degradation is a cooperative enzymatic reaction. This process is characterized by three parameters: the proportion between the rate of production and the maximum potential degradation rate *γ* (corresponding to the maximum reaction velocity in the Michaelis–Menten kinetic); the enzyme saturation threshold *θ* (corresponding to the Michaelis–Menten constant); and the Hill cooperativity coefficient *α* (that would be 1 in a standard uncooperative Michaelis–Menten reaction). During the onset of the replicative senescence we observe a reduction of the velocity ratio *γ*, while the concentration threshold *θ* and the cooperativity coefficient *α* remain constant. Moreover the value of *α* is significantly larger than 1, justifying the choice to include cooperativity in the model.

From a kinetic point of view this can be interpreted as a competitive inhibition mechanism, in which the enzyme active site is blocked by an inhibitor, similar to the usual substrate, that prevents it from properly working. The presence of the inhibitor reduces the capability of the enzyme to convert the substrate into the final product (the *γ* parameter, corresponding to the maximum velocity of the Michaelis–Menten reaction). We remark that the strongly nonlinear change in the nuclear protein amount, as observed experimentally, is caused by an almost linear change in the model parameters. This implies that the strong change in the average nuclear protein amount is not subsequent of clear change in the behavior of the cell, but rather a consequence of small variations amplified by the nonlinearity of the degradation mechanism. Given that the reaction under consideration is the degradation of proteins by the proteasome, an increase of the concentration of inhibitor(s) would lead to a slowdown of protein turnover, driving a further accumulation of inhibitors.

Recent biochemical studies support our results that proteasome activity in cell might be affected upon ageing because of the accumulation of inhibitors instead of a degeneration of proteasome activity [52, 53]. Such an inhibitory effect seems to be tackled in some cell types by varying proteasome isoform content [54], which differ in their kinetics from a quantitative point of view [55].

The results from our analysis, combined with the preexisting experimental and theoretical knowledge, suggest that the accumulation of proteins in the cell nucleus can be described with a good approximation as a reduction of the proteasome activity, due to the accumulation of inhibitors. These inhibitors are probably non–correctly degradated protein debris that drive a vicious cycle that prevents the degradation cycle from working properly, leading to the observed protein accumulation.

We believe that our approach is innovative for the following reasons:

1. We utilized a CME for the description of this process and characterized the stationary distribution as a generalized negative binomial distribution
2. Variations in the parameters distribution are able to discriminate between different stages of cellular senescence
3. Our modeling, also verified by experimental data, are supporting the hypothesis of molecular clogging versus the cellular clock.

In conclusion we think that stochastic modeling of biological processes is a very informative approach, especially if compared with experimental data, because it can shed new light on the complexity of biological processes.

## Materials and Methods

### Culture Conditions, Staining and Quantitative Imaging

Primary adult mouse tail fibroblasts (MTF) were obtained from tail biopsies of 8–12 week old C57 mice as described [56]. MTF were cultured in Dulbecco’s Modified Eagle’s Medium (DMEM) supplemented with 10% FBS in an atmosphere of 2.5% O2, 5% CO2. In contrast to the commonly Mouse Embryo Fibroblast (MEF) cells, MTFs undergo senescence even under physiological oxygen levels, and display characteristic increases of SA-*β*-Gal and senescence regulators such as *p*16^*Ink*4*a*^[57].

MTFs were routinely sub-cultured at 1:4 dilution upon reaching 80% confluence: under these conditions cells reached senescence at passage 12. At each passage cells were seeded onto glass cover slips and fixed as previously described [58], then processed either for the SA-*β*-Gal assay [59] or protein staining and confocal microscopy as described in detail by De Cecco et al. [11].

The data used in the analysis were obtained from a software evaluation of the integral fluorescence signal and the surface extension of the cellular nucleus.

### Numerical simulation

The stationary distribution of the proposed model cannot be written in a closed form for a generic, real valued *α*, although it is possible for integer valued *α* as a function of Pochhammer symbols. We chose to perform a numerical evaluation of the equilibrium solution calculating explicitly the recurrence equation in Eq 4 as a logarithmic equation, limiting the state space to a maximum *n*. This method is fast and accurate enough for a number of molecules in the order of thousands. The accuracy has been tested confronting the results with an expression obtained by symbolic algebra system.

The value *P*_0_ used for the normalization in Eq 7 is then normalized to the sum of the first *n* states, where *n* is the chosen product limit:

$$P_{0}={\left(\sum_{k=1}^{n}\prod_{i=1}^{k}\frac{g_{i-1}}{r_{i}}\right)}^{-1}$$

It is possible to normalize the value of *n* to any multiplicative constant, the only effect being an approximation due to the integer nature of the state space and an equivalent scaling on the constant *θ*. This has been used to rescale the obtained data into the interval [0, 1000], which was the largest interval on which the correctness of the numeric evaluation has been tested. The value of 1000 has been assigned to the highest observed value among all experiments and every other value has been scaled accordingly.

### Parameter estimation by Bayesian MonteCarlo Markov Chain

The parameters for each passage have been evaluated independently from the others by means of an estimation of the likelihood function of the master equation given the data. The likelihood function was evaluated by a MonteCarlo Markov Chain, and the local maximum was taken as the best value (the maximum likelihood). The likelihood function was normalized as probability density function to evaluate the uncertainties of the parameters and their correlation. This is equivalent to a Bayesian analysis of the data with a flat, non-proper prior on all the parameters.

The MonteCarlo Markov Chain was performed with an adaptive Metropolis algorithm to account for the parameters correlation, and the simulation was run for 2 ⋅ 10^5^ steps, with a burn–in period of 10^5^ steps, and thinning factor of 10 (9 out of 10 steps are discarded to reduce sampling correlation).

**Fig 9:**
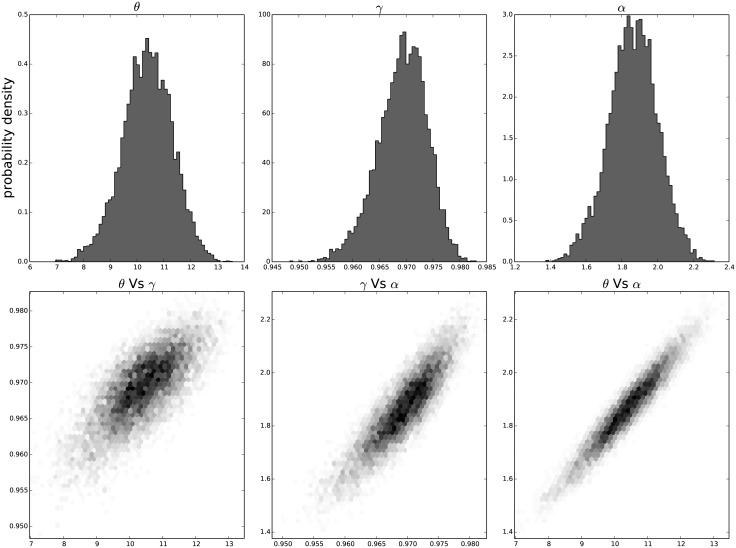
The distribution of the parameters (*α*, *θ* and *γ*) for the third passage estimated with bayesian analysis. In the first line we have the distribution of each parameter. In the second line we have the joint probability distribution of each couple. This show that the values are correlated but without strong non–linearity.

**Fig 10:**
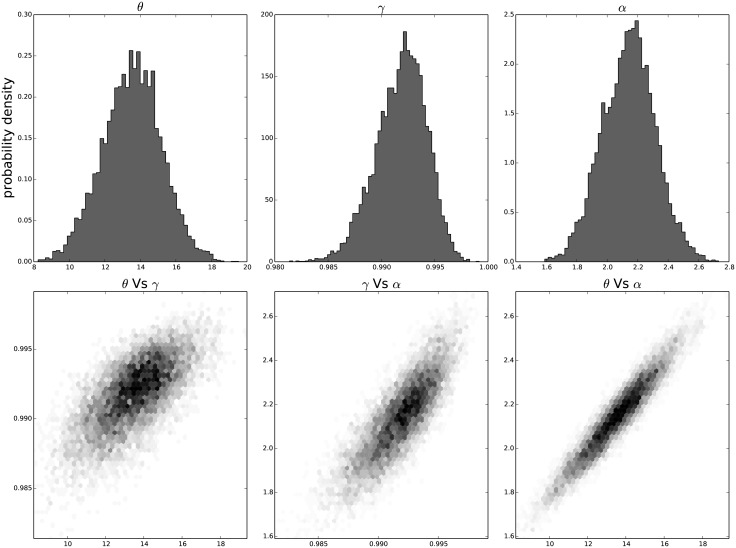
The distribution of the parameters (*α*, *θ* and *γ*) for the ninth passage estimated with bayesian analysis. In the first line we have the distribution of each parameter. In the second line we have the joint probability distribution of each couple. This show that the values are correlated but without strong non–linearity.

**Fig 11:**
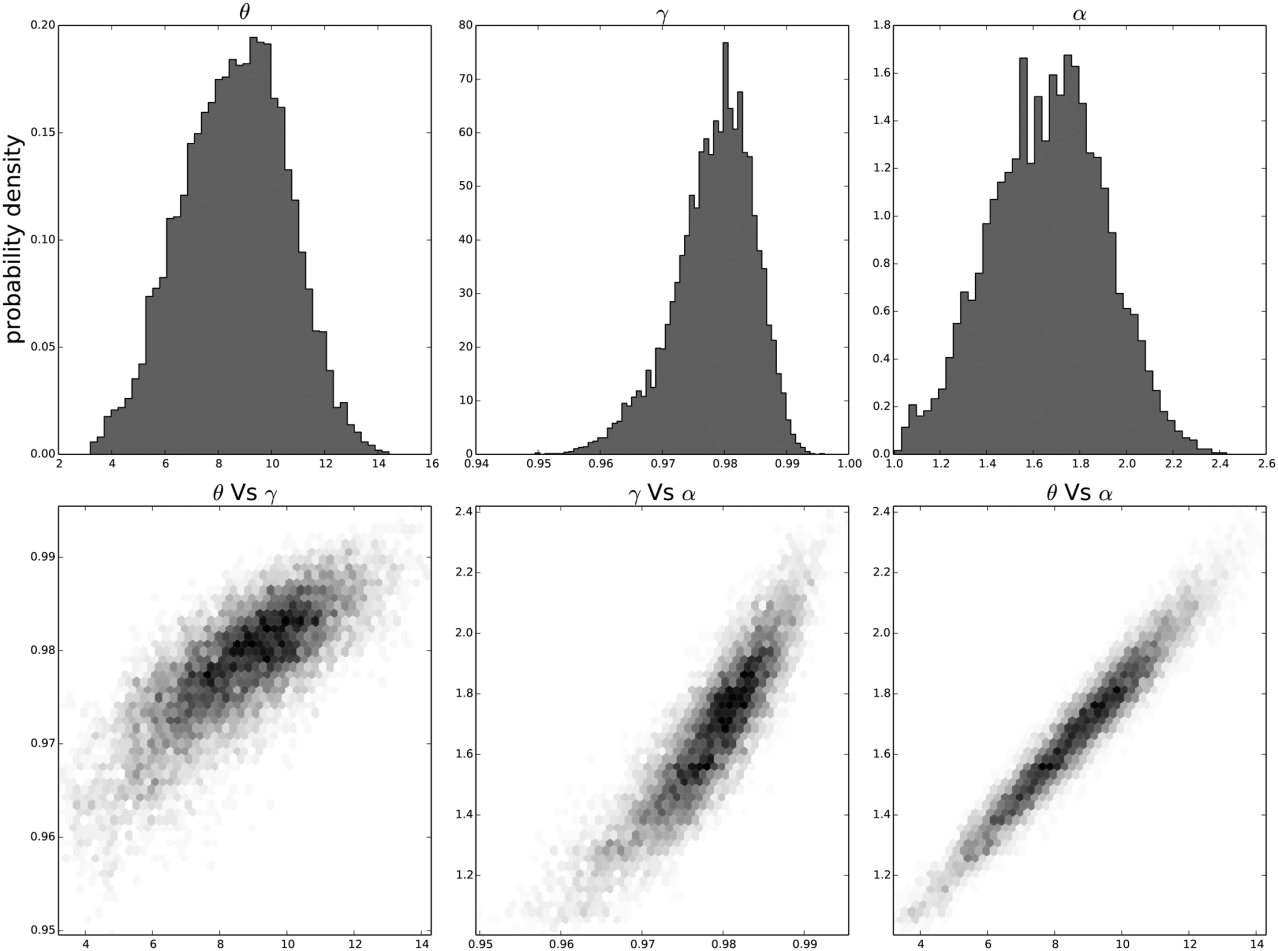
The distribution of the parameters (*α*, *θ* and *γ*) for the tenth passage estimated with bayesian analysis. In the first line we have the distribution of each parameter. In the second line we have the joint probability distribution of each couple. This show that the values are correlated but without strong non–linearity.

**Fig 12:**
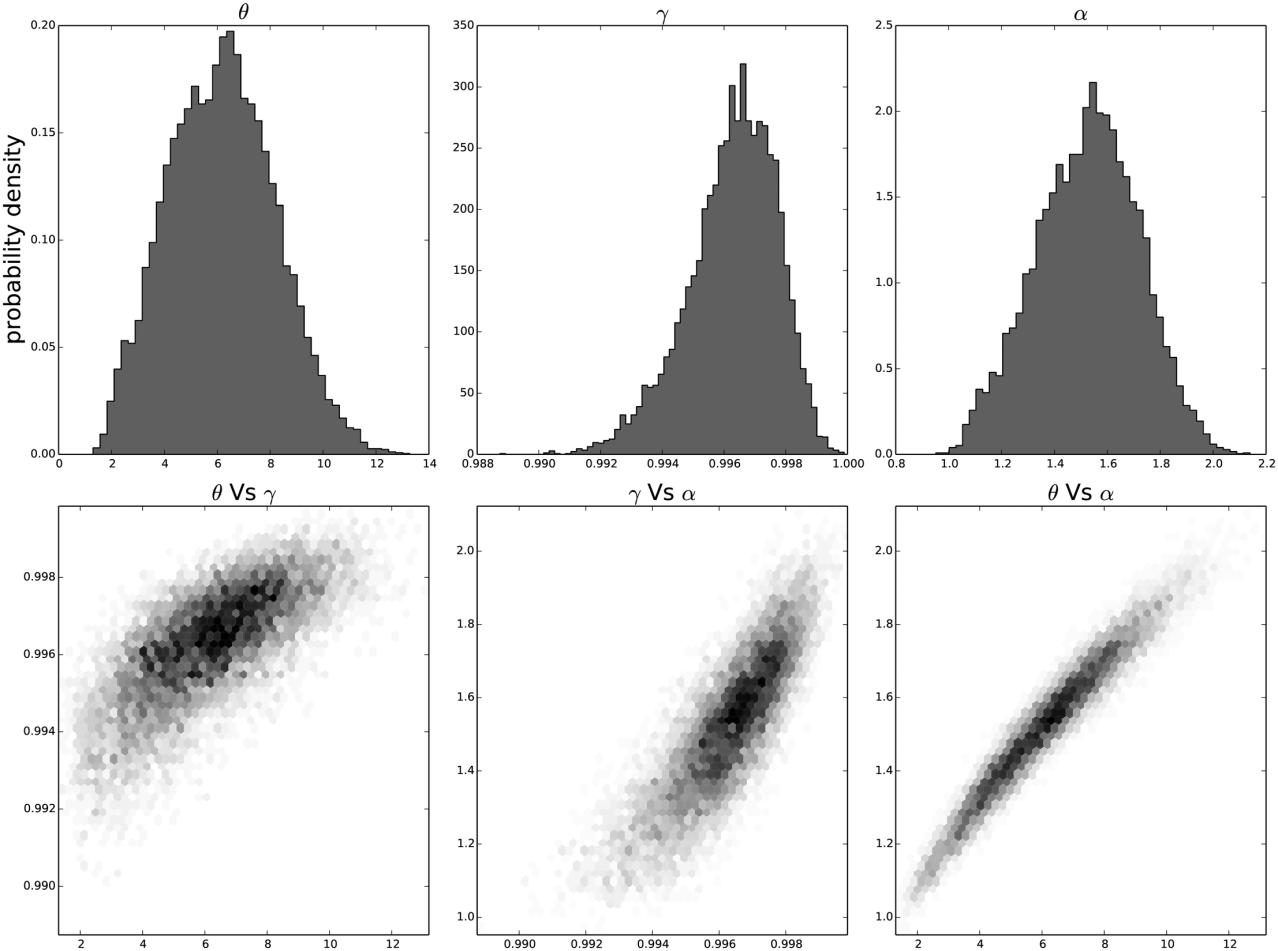
The distribution of the parameters (*α*, *θ* and *γ*) for the eleventh passage estimated with bayesian analysis. In the first line we have the distribution of each parameter. In the second line we have the joint probability distribution of each couple. This show that the values are correlated but without strong non–linearity.

**Fig 13:**
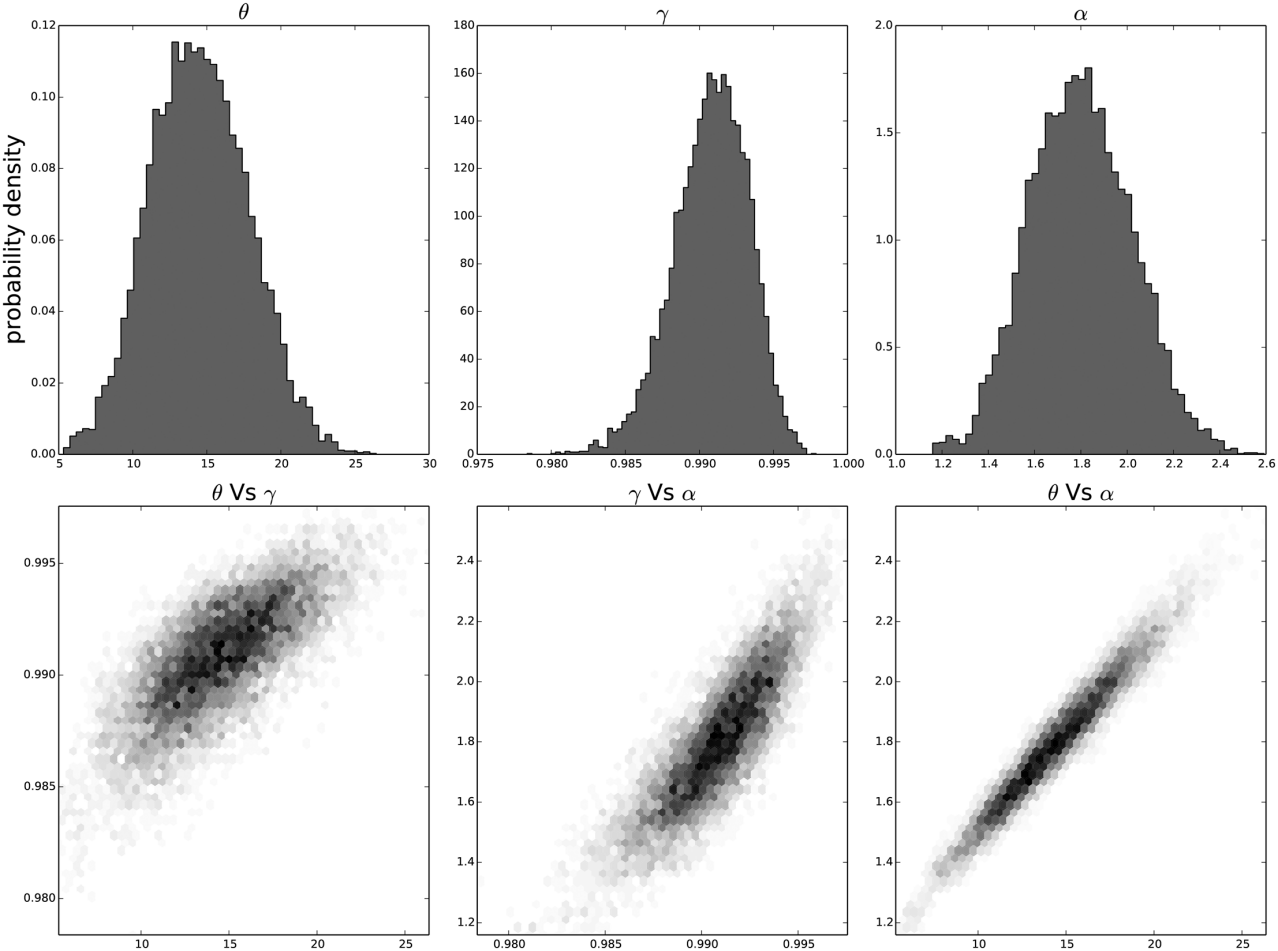
The distribution of the parameters (*α*, *θ* and *γ*) for the twelfth passage estimated with bayesian analysis. In the first line we have the distribution of each parameter. In the second line we have the joint probability distribution of each couple. This show that the values are correlated but without strong non–linearity.

**Fig 14:**
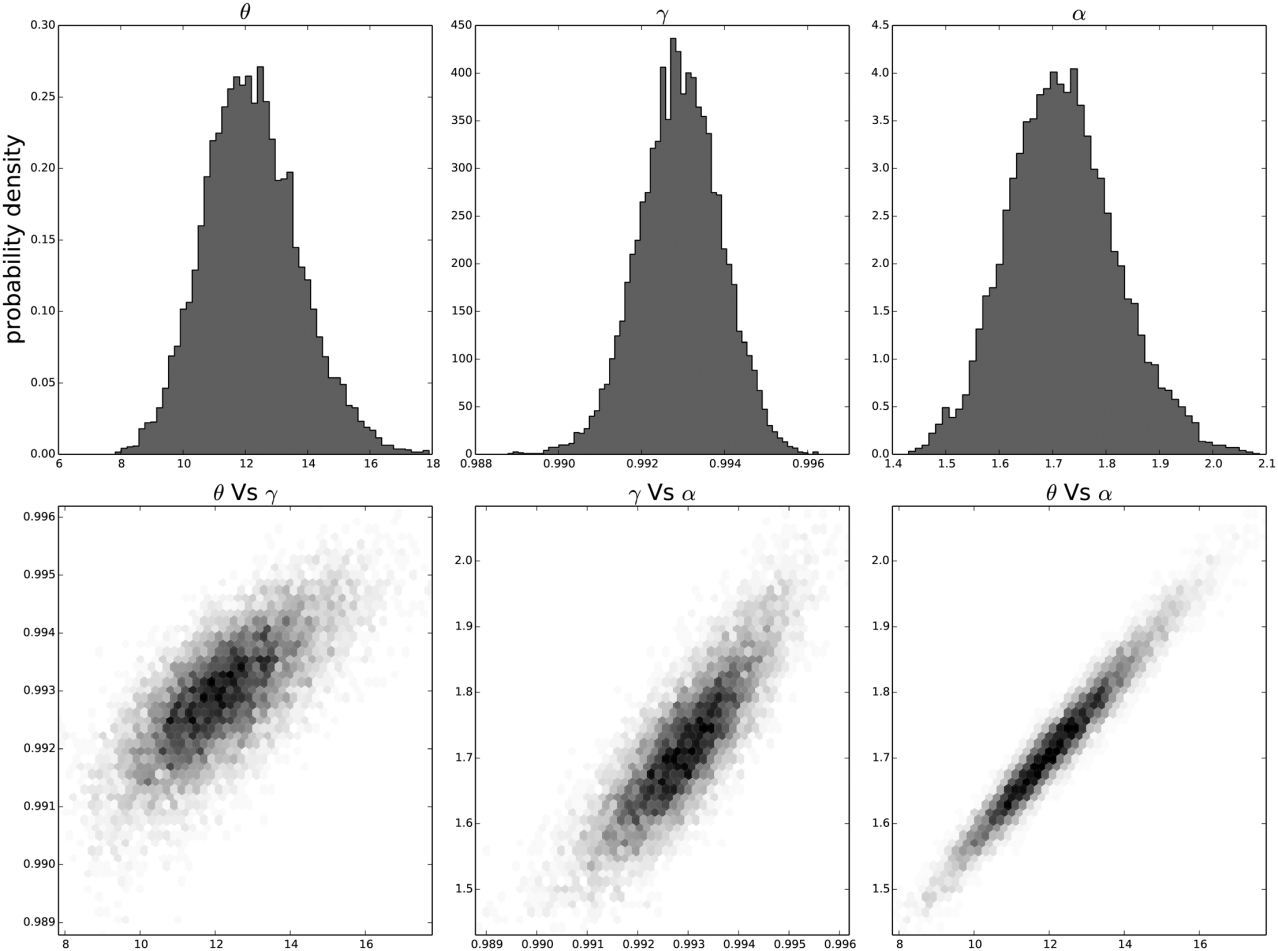
The distribution of the parameters (*α*, *θ* and *γ*) for the thirthinth passage estimated with bayesian analysis. In the first line we have the distribution of each parameter. In the second line we have the joint probability distribution of each couple. This show that the values are correlated but without strong non–linearity.

The resulting distribution of the individual parameters and their joint distribution for each passage in time can be seen in Fig 9 for the results on the third passage data, Fig 10 for the results on the ninth passage data, Fig 11 for the results on the tenth passage data, Fig 12 for the results on the eleventh passage data, Fig 13 for the results on the twelfth passage data, Fig 14 for the results on the thirthinth passage data. These figures show that the parameters have a good behavior, close to a Normal distribution. There is a correlation among the parameters’ value, but there are no non-linearities and the parameters’ space appears well mixed. These results confirm that the parameter estimation has been successfully performed without any biases.

For each parameter a linear trend, as a function of the replicative passages, was estimated with a weighted linear regression, represented by the dashed gray line. The light gray region represents the 95% uncertainty margin for the linear trend, and gives a graphical representation of the significance of the linear trend.

To perform the Bayesian estimation the library pymc [60] of the python [61] language has been used, together with the libraries numpy [62], scipy [63], matplotlib [64], sympy [65] and pandas [66], using the ipython [67] environment.

### Bootstrap significativity computation

We tested the predictive power of our model by fitting the experimentally measured distribution of nuclear protein amount and comparing the goodness of fit of the resulting distribution with the Negative Binomial. For each distribution we evaluated a p-value for the null hypothesis that the distribution family is able to fit all the experimental data. The p-value was evaluated with a bootstrap method. This method adapts a given distribution to the experimental data with a maximum likelihood method and performs an *r* ^2^ to assess the goodness of fit; then the fitted distribution, by a sampling procedure, is used to generate a new set of data of the same size of the original one and performs a new fit and *r* ^2^ estimate on this sample.

By iteratively repeating this procedure we can estimate the expected distribution of the *r* ^2^ and compare the observed value to it, obtaining a value of probability of observing the given value of the test. This can be used as an indicator of the overall goodness of fit of the distribution family, avoiding test distortion due to the fit procedure and data transformation [51].

### Value of *C*_0_ in the Michaelis-Menten degradation

Here we show how the normalization constant *C*_0_ for the standard negative binomial is obtained from Eq 3.

Starting from the recurrence relation we obtain that:

Pn=P0(θ+n)!n!θ!γn=C0(θ+nn)γn

we wish to calculate the total normalized probability:

1=∑n=0∞Pn=C0∑n=0∞(θ+nn)γn

remembering the negative rule of the binomial transformation:

(n+r-1n)=(-1)n(-rn)

and the expansion:

∑n=0∞(rn)xn=(1+x)r

we can substitute and obtain

C0-1=∑n=0∞γn((θ+1)+n-1n)=∑n=0∞-γn(-(θ+1)n)=(1-γ)-(θ+1)

that is the normalization value we use in Eq 5

## Supporting Information

S1 Table This table contains the data used in this work. Each row represents the data from a single cell in each one of the perfomed experiments. The first column contains the size of the cellular nucleus, estimated with fluorescent probing. The second column contains the raw value of the fluorescence signal returned by the microscope. The third column represent the replicative passage of the observation. The fourth column represent which replication of the experiment was used for the observation.
(PDF)
