# Supplementary material for: Active Degradation Explains the Distribution of Nuclear Proteins during Cellular Senescence
Source: PLoS One. 2015 Jun 26;10(6):e0118442. doi: 10.1371/journal.pone.0118442 (PMC4483236; doi:10.1371/journal.pone.0118442)
Supplement: S1 Table — Each row represents the data from a single cell in each one of the perfomed experiments. The first column contains the size of the cellular nucleus, estimated with fluorescent probing. The second column contains the raw value of the fluorescence signal returned by the microscope. The third column represent the replicative passage of the observation. The fourth column represent which replication of the experiment was used for the observation. (PDF) [file pone.0118442.s001.pdf]

Active Degradation Explains the Distribution of Nuclear Proteins during Cellular Senescence

Supplementary Materials

Enrico Giampieri[1,*], Marco De Cecco[2], Daniel Remondini[1], John Sedivy[2], Gastone Castellani[1]
1 Department of Physics and Astronomy, Bologna University, Bologna, Italy and INFN Bologna.
2 Department of Molecular Biology, Cell Biology and Biochemistry, Center for Genomics and Proteomics, Brown University, Providence, RI, USA.
∗ E-mail: enrico.giampieri@unibo.it

Supplementary Table 1

NUCLEUS AREA	FUORESCENT SIGNAL	PASSAGE NUMBER	EXPERIMENT ID
11505	3971	13	4
8590	4610	13	4
10348	4130	13	4
10166	3193	13	4
20864	10877	13	4
10919	2579	13	4
10535	5004	13	4
11266	5956	13	4
8143	1593	13	4
9067	3141	13	4
10655	3660	13	4
7316	1519	13	4
6433	1917	13	4
9898	3981	13	4
14172	7244	13	4
13397	5059	13	4
15614	3452	13	4
12084	3835	13	4
6547	2411	13	4
14267	4115	13	4
10707	4222	13	4
9220	2386	13	4
2977	611	13	4
8109	1533	13	4
3061	597	13	4
3022	411	13	4
8749	3500	13	4
5938	2048	13	4
7017	2033	13	4
13853	5427	13	4
8118	2045	13	4
3618	1126	13	4

NUCLEUS AREA	FUORESCENT SIGNAL	PASSAGE NUMBER	EXPERIMENT ID
11800	5399	13	4
18236	5946	13	4
32649	14974	13	4
10481	3842	13	4
9772	4120	13	4
9717	3699	13	4
35916	11497	13	4
10074	2622	13	4
7830	2172	13	4
7331	2026	13	4
15321	3042	13	4
17064	6463	13	4
10967	4479	13	4
20360	10145	13	4
5914	2506	13	4
6065	3443	13	4
8336	2601	13	4
18315	8105	13	4
14586	11426	13	4
7831	3071	13	4
8598	2029	13	4
6208	2330	13	4
10925	3098	13	4
9369	2856	13	4
17124	7820	13	4
9916	2471	13	4
8805	2098	13	4
9362	1769	13	4
9042	2130	13	4
13330	3810	13	4
8110	3464	13	4
7116	1650	13	4
18189	4264	13	4
5129	1521	13	4
5615	1537	13	4
11648	6834	13	4
11493	5881	13	4
9216	2661	13	4
11573	2707	13	4
7796	1717	13	4
16771	10837	13	4
7336	1996	13	4
8279	2766	13	4
15369	5330	13	4
9798	3869	13	4
5595	2186	13	4
22634	10094	13	4
18243	13400	13	4

NUCLEUS AREA	FUORESCENT SIGNAL	PASSAGE NUMBER	EXPERIMENT ID
22013	9148	13	4
6618	1688	13	4
3991	1076	13	4
9154	5149	13	4
7391	2575	13	4
9201	3181	13	4
15244	4092	13	4
4136	1556	13	4
3939	2568	13	4
2979	2525	13	4
10663	3586	13	4
9385	3078	13	4
17024	5506	13	4
8499	2229	13	4
6981	1810	13	4
18032	4606	13	4
7401	1774	13	4
5709	751	13	4
7715	1072	13	4
15446	2790	13	4
11315	3389	13	4
5477	779	13	4
8004	1389	13	4
7196	2149	13	4
6770	2559	13	4
7197	2809	13	4
11879	5909	13	4
11958	6323	13	4
5285	2683	13	4
11817	4663	13	4
11092	2815	13	4
11369	8005	13	4
6424	1301	13	4
4230	285	13	4
8383	2443	13	4
14280	6056	13	4
7864	2137	13	4
6929	1435	13	4
9855	2466	13	4
3639	1198	3	1
3322	880	3	1
1531	509	3	1
4590	1806	3	1
3800	1426	3	1
2448	1193	3	1
2236	947	3	1
3202	1512	3	1
1586	865	3	1

NUCLEUS AREA	FUORESCENT SIGNAL	PASSAGE NUMBER	EXPERIMENT ID
4904	2037	3	1
3834	799	3	1
3667	1071	3	1
4386	1195	3	1
3788	1156	3	1
2741	1373	3	1
4307	1688	3	1
2681	1023	3	1
1845	895	3	1
2789	1237	3	1
3775	1686	3	1
4126	1967	3	1
1285	332	3	1
4620	1127	3	1
3749	1129	3	1
2584	990	3	1
2577	961	3	1
3642	1620	3	1
2865	1402	3	1
2111	1078	3	1
3844	1705	3	1
2623	1229	3	1
1572	857	3	1
1686	356	3	1
2394	622	3	1
2834	854	3	1
3281	974	3	1
5039	1938	3	1
3659	1535	3	1
4503	1910	3	1
3276	851	3	1
1907	555	3	1
3833	1036	3	1
5049	1445	3	1
5011	1524	3	1
3681	1306	3	1
3127	1373	3	1
1645	659	3	1
2512	1228	3	1
1586	620	3	1
3835	1487	3	1
3782	1822	3	1
1399	468	3	1
1948	427	3	1
4500	1267	3	1
3232	908	3	1
5066	1350	3	1
3173	1355	3	1

NUCLEUS AREA	FUORESCENT SIGNAL	PASSAGE NUMBER	EXPERIMENT ID
4284	1728	3	1
4114	1645	3	1
4206	1058	3	1
2120	583	3	1
2071	643	3	1
3926	1220	3	1
4800	1543	3	1
2867	903	3	1
2002	807	3	1
2530	981	3	1
3548	1296	3	1
4664	1685	3	1
2373	932	3	1
1488	623	3	1
3809	1113	3	1
3625	906	3	1
1534	624	3	1
2566	888	3	1
3666	1163	3	1
2222	877	3	1
2539	958	3	1
3277	1005	3	1
3883	1165	3	1
2487	1286	3	1
2736	1126	3	1
3612	1493	3	1
2788	1091	3	1
2295	1041	3	1
2381	618	3	1
4146	1256	3	1
1815	484	3	1
2600	995	3	1
2899	1123	3	1
2874	1116	3	1
2466	1155	3	1
4204	1581	3	1
6380	2387	3	1
2721	326	3	1
4358	560	3	1
1881	378	3	1
4844	837	3	1
3886	719	3	1
5678	1438	3	1
1998	802	3	1
3249	784	3	1
3769	573	3	1
2589	997	3	1
2897	840	3	1

NUCLEUS AREA	FUORESCENT SIGNAL	PASSAGE NUMBER	EXPERIMENT ID
2901	807	3	1
4585	526	3	1
7520	808	3	1
1807	579	3	1
1661	559	3	1
3468	561	3	1
2379	766	3	1
2011	753	3	1
1642	621	3	1
1997	715	3	1
2513	536	3	1
3996	480	3	1
2243	190	3	1
4198	452	3	1
4440	1055	3	1
4437	1082	3	1
2100	847	3	1
2242	700	3	1
4857	1120	3	1
3127	858	3	1
3069	880	3	1
3982	1018	3	1
3289	441	3	1
2259	863	3	1
4128	829	3	1
3212	794	3	1
2658	727	3	1
5037	1133	3	1
6467	1831	3	1
1393	519	3	1
3249	759	3	1
3440	837	3	1
3671	903	3	1
2359	630	3	1
4235	921	3	1
8217	852	3	1
2674	282	3	1
1264	133	3	1
4819	622	3	1
4968	1000	3	1
3688	767	3	1
4162	866	3	1
4409	933	3	1
2687	638	3	1
2363	687	3	1
3514	907	3	1
1844	601	3	1
1355	152	3	1

NUCLEUS AREA	FUORESCENT SIGNAL	PASSAGE NUMBER	EXPERIMENT ID
2336	789	3	1
2515	316	3	1
2390	710	3	1
3241	653	3	1
2316	594	3	1
4346	1003	3	1
1862	554	3	1
1938	209	3	1
1378	161	3	1
3201	370	3	1
3980	660	3	1
2550	854	3	1
1940	691	3	1
1823	624	3	1
2898	1109	3	1
3978	1249	3	1
1811	652	3	1
3838	1106	3	1
2907	1135	3	1
3431	1195	3	1
4213	957	3	1
3184	911	3	1
4485	1293	3	1
3200	848	3	1
1344	492	3	1
2706	775	3	1
2645	864	3	1
2386	675	3	1
2185	775	3	1
4446	841	3	1
3188	1010	3	1
2762	770	3	1
2538	511	3	1
2521	516	3	1
2714	621	3	1
1697	405	3	1
2799	555	3	1
1919	377	3	1
1484	327	3	1
2335	482	3	1
2380	412	3	1
1862	412	3	1
1630	420	3	1
4060	1021	3	1
3686	1107	3	1
3113	1198	3	1
2949	888	3	1
3073	1088	3	1

NUCLEUS AREA	FUORESCENT SIGNAL	PASSAGE NUMBER	EXPERIMENT ID
2500	722	3	1
1932	618	3	1
1864	581	3	1
2900	1036	3	1
4054	1169	3	1
1901	624	3	1
3348	836	3	1
2084	734	3	1
2178	787	3	1
3467	1252	3	1
1910	817	3	1
2363	374	3	1
1746	400	3	1
3158	698	3	1
1791	531	3	1
7282	2096	3	1
3395	853	3	1
5929	1370	3	1
3263	1087	3	1
3253	620	3	1
2509	759	3	1
2303	688	3	1
4831	1234	3	1
1509	468	3	1
2305	787	3	1
2094	717	3	1
1687	383	3	1
2125	508	3	1
2507	824	3	1
3174	1069	3	1
1337	450	3	1
1396	472	3	1
2112	709	3	1
3047	1065	3	1
1264	465	3	1
2464	634	3	1
3491	1081	3	1
1688	557	3	1
1603	571	3	1
2443	767	3	1
3064	1225	3	1
2840	1083	3	1
2941	1073	3	1
6078	2158	3	1
3375	1270	3	1
1854	550	3	1
1639	496	3	1
1864	549	3	1

NUCLEUS AREA	FUORESCENT SIGNAL	PASSAGE NUMBER	EXPERIMENT ID
1562	543	3	1
2443	776	3	1
1839	545	3	1
5575	1554	3	1
2469	662	3	1
3389	1145	3	1
3585	1280	3	1
4047	1322	3	1
1820	634	3	1
6163	2136	3	1
4436	1503	3	1
2316	373	3	1
2232	465	3	1
2301	537	3	1
5224	1153	3	1
1800	516	3	1
3548	1133	3	1
1747	490	3	1
2912	734	3	1
2672	703	3	1
2650	929	3	1
4024	1304	3	1
1499	678	3	1
2113	817	3	1
2980	602	3	1
3592	910	3	1
3295	940	3	1
2849	744	3	1
2067	652	3	1
3471	937	3	1
2662	839	3	1
1979	682	3	1
1397	575	3	1
2045	441	3	1
2809	683	3	1
2645	754	3	1
3023	1028	3	1
2674	881	3	1
2103	917	3	1
1636	621	3	1
2920	1154	3	1
1403	599	3	1
2669	1127	3	1
2749	930	3	1
1955	747	3	1
2103	767	3	1
2670	1185	3	1
1339	725	3	1

NUCLEUS AREA	FUORESCENT SIGNAL	PASSAGE NUMBER	EXPERIMENT ID
2127	748	3	1
2652	678	3	2
2114	680	3	2
3291	615	3	2
2899	651	3	2
3479	1010	3	2
3908	1159	3	2
4536	899	3	2
2836	655	3	2
5439	1234	3	2
1449	240	3	2
5382	897	3	2
11818	2324	3	2
3578	553	3	2
3576	620	3	2
11936	2545	3	2
2678	430	3	2
2576	899	3	2
4804	1297	3	2
2485	691	3	2
2580	759	3	2
12022	2821	3	2
2952	553	3	2
3071	773	3	2
2497	828	3	2
8104	1654	3	2
2408	601	3	2
2490	601	3	2
2671	641	3	2
10598	2030	3	2
5718	750	3	2
11118	2117	3	2
2714	632	3	2
3525	510	3	2
2640	633	3	2
2923	511	3	2
3645	1732	3	2
9754	2275	3	2
2022	611	3	2
2980	869	3	2
3404	661	3	2
3048	868	3	2
4058	1140	3	2
3480	817	3	2
2957	687	3	2
2685	477	3	2
3380	927	3	2
1552	257	3	2

NUCLEUS AREA	FUORESCENT SIGNAL	PASSAGE NUMBER	EXPERIMENT ID
2739	630	3	2
2666	555	3	2
5815	902	3	2
4910	1003	3	2
2689	587	3	2
3107	609	3	2
3182	809	3	2
2175	713	3	2
3952	1630	3	2
2827	710	3	2
2923	743	3	2
2755	774	3	2
2257	740	3	2
2802	724	3	2
2633	780	3	2
3453	706	3	2
3164	538	3	2
2706	572	3	2
2544	596	3	2
4974	981	3	2
3893	1107	3	2
2662	640	3	2
7447	1269	3	2
4472	1383	3	2
3655	1139	3	2
2735	788	3	2
4325	853	3	2
3020	764	3	2
6050	1501	3	2
3878	1190	3	2
2338	885	3	2
3489	994	3	2
3634	609	3	2
5612	1203	3	2
5061	692	3	2
2557	601	3	2
3288	618	3	2
3946	1097	3	2
3891	717	3	2
3308	592	3	2
5235	936	3	2
2635	988	3	2
2883	783	3	2
2671	727	3	2
3506	1170	3	2
4225	1490	3	2
4156	997	3	2
4910	1339	3	2

NUCLEUS AREA	FUORESCENT SIGNAL	PASSAGE NUMBER	EXPERIMENT ID
2211	810	3	2
1486	569	3	2
2477	735	3	2
4964	972	3	2
2819	580	3	2
3349	644	3	2
3360	528	3	2
3954	803	3	2
3332	467	3	2
5359	1039	3	2
8528	3799	3	2
3355	951	3	2
5520	1616	3	2
2172	791	3	2
2497	536	3	2
3384	887	3	2
4817	1410	3	2
2849	1063	3	2
3091	701	3	2
3881	999	3	2
2657	848	3	2
3840	772	3	2
2770	705	3	2
3437	568	3	2
3216	506	3	2
3205	446	3	2
3762	751	3	2
3450	1369	3	2
3125	1041	3	2
3025	1013	3	2
2422	913	3	2
2646	658	3	2
2390	952	3	2
6618	2075	3	2
3797	1197	3	2
3121	670	3	2
3189	524	3	2
3352	554	3	2
2869	570	3	2
2851	550	3	2
5339	1087	3	2
4731	1424	3	2
2618	731	3	2
3421	1100	3	2
3420	949	3	2
4934	1221	3	2
2501	675	3	2
2493	672	3	2

NUCLEUS AREA	FUORESCENT SIGNAL	PASSAGE NUMBER	EXPERIMENT ID
2868	852	3	2
3525	688	3	2
3509	614	3	2
11627	2775	3	2
4896	2312	3	2
2945	877	3	2
2639	756	3	2
3137	855	3	2
2456	835	3	2
2572	691	3	2
2822	998	3	2
1819	577	3	2
3419	534	3	2
3376	433	3	2
2728	729	3	2
1663	676	3	2
2430	729	3	2
2616	782	3	2
2502	773	3	2
2859	1080	3	2
7807	2395	3	2
3060	656	3	2
3437	953	3	2
2658	795	3	2
3068	886	3	2
2069	685	3	2
6423	2199	3	2
3113	872	3	2
2082	677	3	2
2645	817	3	2
2568	760	3	2
1731	577	3	2
2756	677	3	2
5034	1366	3	2
1821	668	3	2
3159	956	3	2
2767	866	3	2
1863	632	3	2
3958	1743	3	2
3256	1161	3	2
3662	903	3	2
2948	743	3	2
2958	773	3	2
2964	941	3	2
2839	899	3	2
2230	805	3	2
2557	994	3	2
3843	1074	3	2

NUCLEUS AREA	FUORESCENT SIGNAL	PASSAGE NUMBER	EXPERIMENT ID
5564	1547	3	3
5145	1339	3	3
5922	2272	3	3
4835	1725	3	3
3625	1335	3	3
6510	2353	3	3
7543	2688	3	3
2339	887	3	3
8034	3255	3	3
1997	805	3	3
5306	1943	3	3
4522	1501	3	3
8840	3352	3	3
8280	3071	3	3
4716	1835	3	3
6848	2461	3	3
6466	2433	3	3
8097	2738	3	3
5904	2037	3	3
4070	1582	3	3
7719	1612	3	3
6183	1418	3	3
4179	1051	3	3
6979	1989	3	3
6809	1741	3	3
8653	2719	3	3
5275	1658	3	3
5082	1726	3	3
5014	1705	3	3
4750	1689	3	3
2845	961	3	3
4229	1472	3	3
3657	927	3	3
5399	1424	3	3
8662	2391	3	3
6410	2082	3	3
3653	1176	3	3
4186	1195	3	3
3031	828	3	3
4046	1391	3	3
4831	1405	3	3
5928	2004	3	3
4621	1651	3	3
3895	1369	3	3
7154	2943	3	3
3944	1353	3	3
4141	1494	3	3
4329	959	3	3

NUCLEUS AREA	FUORESCENT SIGNAL	PASSAGE NUMBER	EXPERIMENT ID
3923	942	3	3
5692	1598	3	3
2608	678	3	3
4446	1252	3	3
4044	1180	3	3
3377	1457	3	3
4213	1210	3	3
3622	1228	3	3
9877	3065	3	3
3048	1226	3	3
7821	2407	3	3
3656	1657	3	3
4433	2282	3	3
4610	1541	3	3
3416	916	3	3
5749	1579	3	3
12269	3755	3	3
11538	5179	3	3
5863	2693	3	3
8869	3331	3	3
4270	1623	3	3
3650	1273	3	3
4111	1164	3	3
4483	1362	3	3
5281	1635	3	3
4421	1524	3	3
3687	1385	3	3
3654	1640	3	3
6196	2641	3	3
2805	1068	3	3
3199	1328	3	3
3832	1630	3	3
2843	913	3	3
3477	1369	3	3
4281	1657	3	3
7727	3026	3	3
2903	851	3	3
7374	2174	3	3
4250	1411	3	3
4587	1506	3	3
3699	1284	3	3
3028	1148	3	3
6017	1868	3	3
2234	815	3	3
3711	1648	3	3
7537	3101	3	3
3750	1509	3	3
3738	1649	3	3

NUCLEUS AREA	FUORESCENT SIGNAL	PASSAGE NUMBER	EXPERIMENT ID
3962	1591	3	3
5289	1781	3	3
4641	1551	3	3
3887	1255	3	3
7861	2638	3	3
3829	1306	3	3
7359	2333	3	3
4298	1646	3	3
4815	1987	3	3
2423	976	3	3
4346	2015	3	3
5696	2953	3	3
10197	4395	3	3
2459	1263	3	3
4407	2112	3	3
3419	1736	3	3
3523	1958	3	3
3428	1467	3	3
2876	1236	3	3
4637	1862	3	3
6920	2561	3	3
2628	1482	3	3
3802	2055	3	3
3065	1505	3	3
3766	1936	3	3
3625	1765	3	3
4516	1984	3	3
4423	2006	3	3
2937	1305	3	3
4258	1918	3	3
3740	1885	3	3
3307	2056	3	3
4642	2204	3	3
2018	1107	3	3
4281	593	3	3
5536	644	3	3
4257	792	3	3
4119	610	3	3
4455	912	3	3
4622	1060	3	3
5385	1271	3	3
4130	827	3	3
5405	882	3	3
5707	908	3	3
6404	954	3	3
3802	793	3	3
4594	740	3	3
5831	1134	3	3

NUCLEUS AREA	FUORESCENT SIGNAL	PASSAGE NUMBER	EXPERIMENT ID
5399	1233	3	3
4698	918	3	3
4248	942	3	3
2977	739	3	3
3123	826	3	3
2727	807	3	3
4455	1006	3	3
5299	1000	3	3
4543	945	3	3
4389	1024	3	3
2791	911	3	3
5405	1170	3	3
6033	1439	3	3
4209	1300	3	3
4248	1096	3	3
5871	1037	3	3
6190	1369	3	3
3840	797	3	3
4319	913	3	3
6751	1885	3	3
4589	1289	3	3
12028	3461	3	3
5589	1488	3	3
2838	889	3	3
1988	421	3	3
5342	1032	3	3
6193	1355	3	3
2299	663	3	3
4132	936	3	3
3283	965	3	3
6494	1360	3	3
3825	794	3	3
4234	1111	3	3
6354	1287	3	3
4615	1101	3	3
3842	948	3	3
2820	765	3	3
4305	1353	3	3
4123	1168	3	3
3254	1060	3	3
4059	1219	3	3
5253	1532	3	3
5273	1088	3	3
6258	1132	3	3
4066	969	3	3
2376	513	3	3
4212	1081	3	3
4952	1254	3	3

NUCLEUS AREA	FUORESCENT SIGNAL	PASSAGE NUMBER	EXPERIMENT ID
4580	1177	3	3
3380	1136	3	3
5575	1465	3	3
5878	1464	3	3
4470	827	3	3
5262	884	3	3
3371	780	3	3
2327	482	3	3
5644	1004	3	3
3844	971	3	3
5237	1128	3	3
5122	1291	3	3
3751	928	3	3
3550	850	3	3
4589	1167	3	3
4785	1136	3	3
3290	939	3	3
4061	1072	3	3
4135	1283	3	3
5348	1457	3	3
2699	673	3	3
4053	1191	3	3
4513	1322	3	3
2796	829	3	3
3348	744	3	3
3874	927	3	3
5770	1383	3	3
3261	818	3	3
3482	915	3	3
2439	666	3	3
4546	1301	3	3
6955	2004	3	3
10836	3042	3	3
5495	1577	3	3
5155	1390	3	3
6598	1571	3	3
4066	940	3	3
8039	2078	3	3
5811	1512	3	3
5290	1775	3	3
3915	1063	3	3
3309	749	3	3
2706	947	3	3
4524	1516	3	3
5060	1603	3	3
4267	1133	3	3
4409	1198	3	3
4546	1262	3	3

NUCLEUS AREA	FUORESCENT SIGNAL	PASSAGE NUMBER	EXPERIMENT ID
4930	1352	3	3
4341	1132	3	3
4475	1286	3	3
3681	923	3	3
4358	700	3	3
8233	1593	3	3
4771	610	3	3
6782	1339	3	3
5583	1236	3	3
5604	1167	3	3
4014	744	3	3
4405	1118	3	3
5267	1612	13	1
2554	998	13	1
3485	1340	13	1
3125	1146	13	1
8012	2625	13	1
2166	1098	13	1
2004	783	13	1
9940	4628	13	1
2081	615	13	1
4306	1157	13	1
2881	939	13	1
2257	1902	13	1
4409	2066	13	1
22000	5962	13	1
5659	1876	13	1
16549	3345	13	1
3743	1701	13	1
2764	423	13	1
2478	1173	13	1
10576	4214	13	1
7616	1633	13	1
4100	2190	13	1
8212	3178	13	1
4999	1097	13	1
7382	1815	13	1
9006	3244	13	1
3290	1022	13	1
2058	1222	13	1
10479	3019	13	1
5635	2228	13	1
3962	736	13	1
9528	3143	13	1
6717	1865	13	1
6866	2305	13	1
7514	2838	13	1
2054	1047	13	1

NUCLEUS AREA	FUORESCENT SIGNAL	PASSAGE NUMBER	EXPERIMENT ID
12160	4833	13	1
2161	596	13	1
5834	2642	13	1
6141	1935	13	1
2336	630	13	1
4124	1883	13	1
3752	2005	13	1
6214	2502	13	1
4626	1725	13	1
11527	4250	13	1
5334	1463	13	1
5419	2031	13	1
4408	2114	13	1
5778	2705	13	1
4846	1944	13	1
6645	2542	13	1
8361	3016	13	1
5818	2413	13	1
4603	1986	13	1
4572	1985	13	1
5921	2002	13	1
7303	3039	13	1
5858	2122	13	1
6864	2189	13	1
3098	1067	13	1
7608	5978	13	1
4927	1952	13	1
5799	1286	13	1
7327	2203	13	1
10780	4736	13	1
2597	947	13	1
3488	1683	13	1
2462	885	13	1
8837	3441	13	1
9360	3283	13	1
3380	1017	13	1
6657	2641	13	1
5833	2245	13	1
9400	3202	13	1
2396	1012	13	1
3449	1107	13	1
3820	1182	13	1
5208	2530	13	1
4507	1991	13	1
5638	2074	13	1
5430	1610	13	1
3452	1290	13	1
9446	2628	13	1

NUCLEUS AREA	FUORESCENT SIGNAL	PASSAGE NUMBER	EXPERIMENT ID
9888	6906	13	1
3492	1699	13	1
4564	2733	13	1
12661	4705	13	1
9305	3668	13	1
4405	1366	13	1
10236	4029	13	1
6149	2839	13	1
3119	1586	13	1
6743	2291	13	1
11502	3305	13	1
3994	1491	13	1
7760	3213	13	1
3698	1724	13	1
3793	1010	13	1
3396	1547	13	1
4583	1668	13	1
11700	4120	13	1
7271	3032	13	1
3914	1333	13	1
5148	2536	13	1
3407	1673	13	1
4122	1595	13	1
3729	1913	13	1
8329	3843	13	1
5245	2363	13	1
8727	3577	13	1
3921	2042	13	1
13928	5171	13	1
5302	1928	13	1
10756	4627	13	1
3912	1586	13	1
3183	1050	13	1
6013	4439	13	1
3737	1738	13	1
5865	1828	13	1
5246	2449	13	1
11077	4665	13	1
7560	3664	13	1
11337	3677	13	1
5419	1504	13	1
7883	3280	13	1
6736	2112	13	1
3049	1755	13	1
5183	2203	13	1
3907	1759	13	1
5800	1765	13	1
3849	1585	13	1

NUCLEUS AREA	FUORESCENT SIGNAL	PASSAGE NUMBER	EXPERIMENT ID
3763	1561	13	1
4617	1789	13	1
6934	2916	13	1
2057	653	13	1
4680	1955	13	1
6952	2752	13	1
5187	2784	13	1
6277	1779	13	1
10728	3774	13	1
2037	1054	13	1
2743	1396	13	1
5015	2660	13	1
2932	1184	13	1
5423	2887	13	1
3681	1586	13	1
3873	1974	13	1
10777	7484	13	1
2395	766	13	1
5375	2393	13	1
10931	5376	13	1
2571	790	13	1
5022	1907	13	1
4968	2250	13	1
4070	2032	13	1
5770	1542	13	1
10270	4639	13	1
3567	1807	13	1
5339	1769	13	1
2656	1389	13	1
8640	3216	13	1
11546	4878	13	1
10063	4413	13	1
10725	4350	13	1
6952	2677	13	1
5630	1684	13	1
5104	2834	13	1
12222	5736	13	1
7094	2378	13	1
10208	4737	13	1
12729	5071	13	1
5624	1720	13	1
9583	5523	13	1
5641	2021	13	1
7265	4365	13	1
10014	3944	13	1
5710	2005	13	1
6369	3670	13	1
4978	2338	13	1

NUCLEUS AREA	FUORESCENT SIGNAL	PASSAGE NUMBER	EXPERIMENT ID
5318	2024	13	1
5768	2745	13	1
4111	2109	13	1
6953	2921	13	1
9126	3615	13	1
6287	2350	13	1
8689	3214	13	1
2168	1327	13	1
6086	2175	13	1
2219	916	13	1
18112	7408	13	1
5044	2411	13	1
5770	2291	13	1
10390	4855	13	1
10096	3020	13	1
4011	1653	13	1
10320	4362	13	1
8144	3759	13	1
3055	1608	13	1
3423	1308	13	1
12044	4964	13	1
7653	3604	13	1
5914	1767	13	1
5469	2256	13	1
6975	2637	13	1
3699	1928	13	1
7287	2433	13	1
10604	3609	13	1
8715	3889	13	1
5663	2163	13	1
8915	3117	13	1
6697	2720	13	1
9391	3279	13	1
5080	2255	13	1
5753	2158	13	1
4566	1949	13	1
7410	3107	13	1
12230	5001	13	1
4261	2371	13	1
5781	2180	13	1
4001	2020	13	1
15575	8114	13	1
7225	3637	13	1
3508	964	13	1
13916	6059	13	1
12669	5819	13	1
4030	1806	13	1
6828	2911	13	1

NUCLEUS AREA	FUORESCENT SIGNAL	PASSAGE NUMBER	EXPERIMENT ID
6736	2231	13	1
3505	1175	13	1
12167	4766	13	1
8602	4794	13	1
6223	2711	13	1
7032	2190	13	1
5714	2284	13	1
5350	1815	13	1
7484	2918	13	1
4125	1834	13	1
5047	2632	13	1
9429	3905	13	1
3556	1496	13	1
8348	3755	13	1
9143	4365	13	1
3757	1324	13	1
9629	3917	13	1
6433	3304	13	1
6810	3062	13	1
6724	2040	13	1
3341	1487	13	1
2500	1822	13	1
5372	3216	13	1
7965	4340	13	1
15701	6557	13	1
5208	1868	13	1
7009	2310	13	1
3818	1857	13	1
5931	2515	13	1
2501	1340	13	1
30634	13794	13	1
3004	1489	13	1
3363	1572	13	1
6878	2815	13	1
6049	2421	13	1
7655	2708	13	1
6717	2157	13	1
4948	2173	13	1
13945	6940	13	1
6132	2710	13	1
8371	3261	13	1
4270	2035	13	1
8413	5219	13	1
8446	3077	13	1
5756	2375	13	1
15810	12045	13	1
11279	7189	13	1
9405	4821	13	1

NUCLEUS AREA	FUORESCENT SIGNAL	PASSAGE NUMBER	EXPERIMENT ID
9199	2929	13	1
5176	3512	13	1
4985	1951	13	1
9751	4133	13	1
7173	3557	13	1
8427	2902	13	1
7178	3536	13	1
4714	2116	13	1
7782	2572	13	1
3841	1739	13	1
7045	2188	13	1
8367	3469	13	1
8049	3233	13	1
5225	1605	13	1
14516	9967	13	1
5120	2199	13	1
4828	2638	13	1
6064	2696	13	1
3836	2192	13	1
2942	1594	13	1
4687	2218	13	1
9867	3687	13	1
3714	1525	13	1
6978	2909	13	1
9312	3959	13	1
4521	2136	13	1
6526	2588	13	1
6104	2415	13	1
4790	2465	13	1
12308	4947	13	1
7861	3840	13	1
5608	2668	13	1
4333	1856	13	1
5295	2915	13	1
7163	3275	13	1
8729	4053	13	1
7854	3773	13	1
7709	3276	13	1
4922	2353	13	1
7825	3310	13	1
6874	2807	13	1
8244	3480	13	1
6508	2347	13	1
8341	3835	13	1
7768	2988	13	1
6015	2352	13	1
7039	3356	13	1
9951	4246	13	1

NUCLEUS AREA	FUORESCENT SIGNAL	PASSAGE NUMBER	EXPERIMENT ID
7161	2274	13	1
8658	4511	13	1
8875	3276	13	1
5687	2896	13	1
6638	4166	13	1
4382	2252	13	1
6121	2960	13	1
9611	4179	13	1
4572	2502	13	1
7081	3393	13	1
6770	3714	13	1
4183	1899	13	1
6509	2697	13	1
5404	2463	13	1
4894	2590	13	1
8124	2805	13	1
5744	2480	13	1
5764	2626	13	1
7807	2668	13	1
5811	2563	13	1
4228	2755	13	1
5406	2703	13	1
5772	2453	13	1
5336	1974	13	1
6412	3266	13	1
4909	2006	13	1
9977	4481	13	1
3261	1228	13	1
6709	2587	13	1
5239	2388	13	1
5197	1840	13	1
6067	3544	13	1
4425	2224	13	1
3451	1749	13	1
7548	3479	13	1
6658	3403	13	1
3446	1443	13	1
5236	2342	13	1
5691	1868	13	1
8576	2997	13	1
3571	3339	13	1
4985	2761	13	1
5360	3339	13	1
5127	3309	13	1
4438	1869	13	1
10659	5474	13	1
5608	2208	13	1
8267	3422	13	1

NUCLEUS AREA	FUORESCENT SIGNAL	PASSAGE NUMBER	EXPERIMENT ID
13437	6868	13	1
8006	3365	13	1
6105	2925	13	1
14515	6712	13	1
4480	2107	13	1
5268	2461	13	1
6590	3701	13	1
6616	2707	13	1
4749	2450	13	1
5539	2295	13	1
5093	2508	13	1
6085	1855	13	1
6804	3050	13	1
4741	2600	13	1
5234	2174	13	1
4655	2415	13	1
2801	1283	13	1
4676	1340	13	1
5554	2179	13	1
6312	2399	13	1
3798	2104	13	1
5906	3258	13	1
4865	2565	13	1
3689	2517	13	1
14263	6261	13	1
8637	5498	13	1
4633	1806	13	1
2111	902	13	1
3853	1637	13	1
6338	3108	13	1
3833	2252	13	1
3374	1975	13	1
5837	3462	13	1
2609	1012	13	1
4872	2438	13	1
4563	2132	13	1
4282	1920	13	1
8161	4964	13	3
8215	5322	13	3
8996	3748	13	3
10733	7040	13	3
7935	4135	13	3
13293	7198	13	3
11065	6332	13	3
7917	4446	13	3
7573	3935	13	3
7497	4372	13	3
16997	11221	13	3

NUCLEUS AREA	FUORESCENT SIGNAL	PASSAGE NUMBER	EXPERIMENT ID
16051	11660	13	3
9135	5333	13	3
8024	4257	13	3
11649	7523	13	3
11954	8276	13	3
9321	5888	13	3
15989	7539	13	3
7160	2248	13	3
7213	3949	13	3
10153	4018	13	3
11799	6096	13	3
10538	6229	13	3
9539	4648	13	3
8833	6170	13	3
13116	7395	13	3
10088	3175	13	3
12215	3997	13	3
11990	3773	13	3
9934	3781	13	3
6749	2468	13	3
12536	5860	13	3
4934	2043	13	3
15606	7884	13	3
7375	3098	13	3
10893	3454	13	3
7139	2493	13	3
12071	4641	13	3
11413	4191	13	3
10546	2339	13	3
7926	2422	13	3
17199	5388	13	3
6824	3108	13	3
12481	4655	13	3
7414	3818	13	3
3987	1992	13	3
5329	2823	13	3
9227	3241	13	3
10647	5219	13	3
12967	6486	13	3
9208	4241	13	3
10107	4069	13	3
22274	14424	13	3
13257	6878	13	3
7972	3763	13	3
14906	8621	13	3
9648	6123	13	3
9066	5505	13	3
13147	8100	13	3

NUCLEUS AREA	FUORESCENT SIGNAL	PASSAGE NUMBER	EXPERIMENT ID
10997	7146	13	3
11424	7563	13	3
8185	5112	13	3
13352	6570	13	3
11754	6235	13	3
8665	4381	13	3
7027	4125	13	3
7229	3612	13	3
4765	2224	13	3
4480	2130	13	3
7659	4568	13	3
8819	5846	13	3
8951	4826	13	3
6788	3692	13	3
12841	6288	13	3
6917	2752	13	3
23365	15419	13	3
8215	5272	13	3
7470	3391	13	3
10023	5548	13	3
9905	4044	13	3
5939	2347	13	3
6831	3793	13	3
6016	2083	13	3
8344	3639	13	3
6412	2788	13	3
26772	13290	13	3
2681	1226	13	3
10970	5757	13	3
7322	3131	13	3
9540	3951	13	3
8158	4407	13	3
9943	4178	13	3
11493	6285	13	3
9688	5000	13	3
8742	5776	13	3
8284	3056	13	3
13590	6269	13	3
12288	6647	13	3
17648	7983	13	3
9748	4386	13	3
9030	4496	13	3
10627	3999	13	3
8580	4385	13	3
7774	3152	13	3
18095	7938	13	3
9141	4403	13	3
9579	5379	13	3

NUCLEUS AREA	FUORESCENT SIGNAL	PASSAGE NUMBER	EXPERIMENT ID
3560	635	9	4
2956	433	9	4
3998	492	9	4
4352	721	9	4
5887	918	9	4
14142	4426	9	4
4290	661	9	4
7826	1850	9	4
3209	445	9	4
4071	834	9	4
6370	1724	9	4
9010	1878	9	4
5407	1139	9	4
5261	1278	9	4
5059	1149	9	4
2923	506	9	4
4369	864	9	4
7726	1465	9	4
9362	1741	9	4
5085	1093	9	4
7966	2251	9	4
3613	777	9	4
10948	2054	9	4
7020	1576	9	4
5517	1011	9	4
8259	1578	9	4
8149	1718	9	4
4771	1133	9	4
9474	2648	9	4
8123	2090	9	4
5102	1100	9	4
9111	2590	9	4
8772	2312	9	4
7764	1742	9	4
5274	983	9	4
5289	1174	9	4
5403	1323	9	4
8186	1490	9	4
3741	615	9	4
7470	2117	9	4
6961	1435	9	4
4848	1021	9	4
4536	925	9	4
7707	1659	9	4
4509	915	9	4
5161	1003	9	4
3132	728	9	4
4507	1121	9	4

NUCLEUS AREA	FUORESCENT SIGNAL	PASSAGE NUMBER	EXPERIMENT ID
7542	1990	9	4
7333	1322	9	4
7204	1708	9	4
6920	1969	9	4
3961	3644	9	4
3280	2090	9	4
5327	1307	9	4
10451	3346	9	4
6214	1036	9	4
1973	338	9	4
4458	910	9	4
8468	1871	9	4
6659	1387	9	4
4662	989	9	4
26592	7104	9	4
4669	1067	9	4
4796	995	9	4
3325	3032	9	4
5300	1080	9	4
6395	1587	9	4
6830	1927	9	4
7801	2087	9	4
5996	1534	9	4
6541	1774	9	4
9296	2761	9	4
8431	3412	9	4
8033	3239	9	4
5592	1273	9	4
3608	907	9	4
8209	1952	9	4
12992	6611	9	4
13404	5248	9	4
5759	1044	9	4
8986	1828	9	4
4329	956	9	4
4407	998	9	4
4780	779	9	4
4587	1173	9	4
5043	893	9	4
4300	730	9	4
6020	1821	9	4
26094	12459	9	4
3476	740	9	4
6542	2379	9	4
6175	2343	9	4
4916	1167	9	4
6838	1236	9	4
5828	1633	9	4

NUCLEUS AREA	FUORESCENT SIGNAL	PASSAGE NUMBER	EXPERIMENT ID
3641	636	9	4
5944	1259	9	4
5572	1440	9	4
4052	879	9	4
4164	920	9	4
6086	1117	9	4
6776	1977	9	4
6733	1794	9	4
8890	2153	9	4
5330	1307	9	4
3578	654	9	4
27410	11234	9	4
4241	1635	9	4
5918	1159	9	4
6519	1231	9	4
8732	3483	9	4
3428	861	9	4
5473	1415	9	4
6989	1820	9	4
5959	998	9	4
4100	688	9	4
4427	935	9	4
2107	449	9	4
5326	975	9	4
6622	1631	9	4
6395	1485	9	4
8765	1879	9	4
4609	1038	9	4
2783	445	9	4
5122	1105	9	4
10530	3762	9	4
6567	2101	9	4
4113	1578	9	4
8412	2004	9	4
5825	1208	9	4
8502	3571	9	4
3325	898	9	4
5365	1555	9	4
9310	2896	9	4
7222	2644	9	4
7140	2634	9	4
9913	3376	9	4
6847	2147	9	4
5068	962	9	4
3889	896	9	4
3732	882	9	4
11411	5374	9	4
5692	1498	9	4

NUCLEUS AREA	FUORESCENT SIGNAL	PASSAGE NUMBER	EXPERIMENT ID
7392	2440	9	4
4424	947	9	4
10641	2460	9	4
5414	1845	9	4
17690	5531	9	4
6692	2089	9	4
6149	1928	9	4
5428	2126	9	4
5650	2215	9	4
7618	1793	9	4
12538	4019	9	4
3136	560	9	4
11963	3295	9	4
11591	4331	9	4
3649	1036	9	4
8523	2344	9	4
8422	2233	9	4
3461	901	9	4
10275	2901	9	4
7610	1566	9	4
4686	1018	9	4
11298	4064	9	4
5315	2107	9	4
8352	1614	9	4
3888	1085	9	4
4610	1326	9	4
3667	980	9	4
7062	2511	9	4
4755	1315	9	4
7315	2566	9	4
7463	1904	9	4
10149	2343	9	4
13882	3979	9	4
6809	1944	9	4
10441	2681	9	4
7169	2242	9	4
8492	3015	9	4
6657	2055	9	4
7243	1846	9	4
8696	2640	9	4
6318	1428	9	4
5822	1664	9	4
12545	4279	9	4
3559	798	9	4
3670	1252	9	4
8507	2189	9	4
4058	1014	9	4
7082	2612	9	4

NUCLEUS AREA	FUORESCENT SIGNAL	PASSAGE NUMBER	EXPERIMENT ID
8422	2035	9	4
5167	1218	9	4
10309	3660	9	4
4767	974	9	4
12374	2949	9	4
6100	1482	9	4
9306	2594	9	4
8827	1503	9	4
4610	1331	9	4
9098	2874	9	4
13417	4294	9	4
3816	1187	9	4
8398	3029	9	4
8156	2906	9	4
7380	2579	9	4
6873	1827	9	4
8449	3366	9	4
2204	564	9	4
6694	2088	9	4
7238	1632	9	4
3241	923	9	4
4156	1267	9	4
5796	1805	9	4
7099	1565	9	4
8563	2116	9	4
7214	1166	9	4
7803	1236	9	4
4832	1477	9	4
7388	1927	9	4
5098	1653	9	4
7244	1117	9	4
7706	1614	9	4
5384	1176	9	4
4158	1886	9	4
5789	1215	9	4
7889	1582	9	4
5179	4175	9	4
5388	1150	9	4
6482	1479	9	4
8574	4513	9	4
7111	3270	9	4
9763	1492	9	4
10398	4022	9	4
10652	4520	9	4
6718	1930	9	4
6202	1866	9	4
7517	1531	9	4
8643	1251	9	4

NUCLEUS AREA	FUORESCENT SIGNAL	PASSAGE NUMBER	EXPERIMENT ID
5921	1385	9	4
6305	1798	9	4
7484	1348	9	4
7484	2670	9	4
7227	1585	9	4
6349	1369	9	4
7756	1475	9	4
6613	1391	9	4
7274	1345	9	4
7237	1306	9	4
6998	673	9	4
3880	487	9	4
2506	432	9	4
2013	478	9	4
6587	2867	12	4
6480	2442	12	4
10500	4698	12	4
4828	1950	12	4
5584	2077	12	4
5868	2626	12	4
6162	2393	12	4
5008	1545	12	4
4714	1194	12	4
4804	1401	12	4
7415	3044	12	4
6398	3965	12	4
3559	2179	12	4
7690	3053	12	4
8004	3766	12	4
8824	4498	12	4
7630	2818	12	4
8412	2935	12	4
7562	3011	12	4
7180	3418	12	4
11970	3536	12	4
10775	4451	12	4
11188	9337	12	4
7794	2889	12	4
4889	1452	12	4
7967	2458	12	4
10157	4749	12	4
3816	1020	12	4
7515	3292	12	4
7968	2854	12	4
7739	4410	12	4
6297	2313	12	4
4469	1505	12	4
7817	5301	12	4

NUCLEUS AREA	FUORESCENT SIGNAL	PASSAGE NUMBER	EXPERIMENT ID
6767	2499	12	4
7703	1771	12	4
9877	1969	12	4
6952	1698	12	4
6019	1747	12	4
9081	4395	12	4
8809	4496	12	4
13983	7660	12	4
4404	1799	12	4
4824	2039	12	4
4572	1785	12	4
5205	2631	12	4
5047	2379	12	4
7627	1914	12	4
11458	4424	12	4
7028	5017	12	4
10472	3908	12	4
13579	9363	12	4
6902	2971	12	4
3667	1867	12	4
7440	3006	12	4
9362	2930	12	4
8139	2596	12	4
11924	4561	12	4
12813	6017	12	4
11423	4012	12	4
10507	6221	12	4
8745	5167	12	4
12066	5272	12	4
4677	1933	12	4
9993	2660	12	4
12090	3963	12	4
8358	1519	12	4
5182	2499	12	4
16061	6950	12	4
12494	7768	12	4
3830	1714	12	4
6534	5963	12	4
6115	4919	12	4
7394	3117	12	4
7381	3075	12	4
4746	1155	12	4
3343	1132	12	4
7725	4362	12	4
14170	4506	12	4
11197	3843	12	4
9445	3974	12	4
18149	3678	12	4

NUCLEUS AREA	FUORESCENT SIGNAL	PASSAGE NUMBER	EXPERIMENT ID
10031	4854	12	4
7568	1886	12	4
23088	6133	12	4
11986	2807	12	4
10144	6217	12	4
11824	4049	12	4
12510	3883	12	4
10663	5556	12	4
5953	1057	12	4
5220	976	12	4
8465	2600	12	4
12172	2413	12	4
7780	2847	12	4
8419	3321	12	4
6451	2025	12	4
8536	1295	12	4
5296	1509	12	4
11334	5977	12	4
9520	2794	12	4
11838	3410	12	4
12862	3340	12	4
4930	730	12	4
6089	885	12	4
4774	1908	12	4
7371	1852	12	4
6638	2026	12	4
7391	2462	12	4
4366	915	12	4
5183	1362	12	4
6741	907	12	4
4479	1061	12	4
8467	2085	12	4
7202	1116	12	4
8484	1855	12	4
4714	2109	12	4
5126	2103	12	4
8341	2292	12	4
7853	1987	12	4
6168	2276	12	4
7197	2643	12	4
6943	2141	12	4
5993	2287	12	4
9204	3030	12	4
6606	2020	12	4
6611	4187	12	4
4317	1508	12	4
5670	1560	12	4
13206	5064	12	4

NUCLEUS AREA	FUORESCENT SIGNAL	PASSAGE NUMBER	EXPERIMENT ID
10919	2885	12	4
8145	3011	12	4
7800	2924	12	4
4140	1701	12	4
12832	6012	12	4
4920	2002	12	4
12590	6083	12	4
4864	3672	12	4
8446	6911	12	4
13365	5515	12	4
3689	1626	12	4
4525	1573	12	4
3415	636	12	4
6378	1599	12	4
14501	5090	12	4
12195	2944	12	4
13558	5885	12	4
12260	5926	12	4
4147	1659	12	4
11448	5584	12	4
5065	1646	12	4
12850	5112	12	4
5294	2124	12	4
5185	2368	12	4
5766	3032	12	4
8069	2593	12	4
3831	1834	12	4
8860	7631	12	4
10695	8972	12	4
6087	2301	12	4
5832	2238	12	4
2719	2703	12	4
8931	2274	12	4
6591	4475	12	4
6540	3724	12	4
9823	3407	12	4
16658	3958	12	4
7166	1936	12	4
3111	2703	12	4
10613	5143	12	4
2452	2334	12	4
7499	2364	12	4
4843	2138	12	4
3993	1610	12	4
10865	2634	12	4
13866	6527	12	4
5210	1292	12	4
11106	2748	12	4

NUCLEUS AREA	FUORESCENT SIGNAL	PASSAGE NUMBER	EXPERIMENT ID
4811	1354	12	4
7996	6001	12	4
10953	2809	12	4
5913	2224	13	2
5743	1829	13	2
21616	14790	13	2
5252	1235	13	2
11428	2834	13	2
7905	2002	13	2
8038	2827	13	2
13460	3188	13	2
7132	2118	13	2
5121	1187	13	2
15214	6856	13	2
10414	2878	13	2
6982	1650	13	2
11152	5320	13	2
8876	1870	13	2
6338	1036	13	2
7621	3291	13	2
7018	1739	13	2
9453	4397	13	2
11590	4257	13	2
9645	5945	13	2
8473	4238	13	2
14826	13119	13	2
9919	4859	13	2
9394	7382	13	2
13025	10806	13	2
13542	11178	13	2
7315	4735	13	2
7039	4623	13	2
9321	6651	13	2
12494	4977	13	2
9467	5341	13	2
16179	9259	13	2
11792	7126	13	2
8960	6089	13	2
11985	5878	13	2
11227	5428	13	2
10192	5427	13	2
6374	2859	13	2
10982	4097	13	2
8693	5323	13	2
10241	4054	13	2
10951	5681	13	2
8305	4974	13	2
15421	8071	13	2

NUCLEUS AREA	FUORESCENT SIGNAL	PASSAGE NUMBER	EXPERIMENT ID
9074	2323	11	4
2409	366	11	4
2984	1033	11	4
1997	646	11	4
2164	484	11	4
2143	506	11	4
9042	1568	11	4
2724	768	11	4
2108	438	11	4
11632	2862	11	4
10330	1789	11	4
12674	2251	11	4
9596	2593	11	4
5006	644	11	4
10102	1668	11	4
12899	2279	11	4
12614	2459	11	4
7057	1057	11	4
23242	5653	11	4
5427	2283	11	4
4419	838	11	4
3903	643	11	4
8471	1582	11	4
20145	6363	11	4
7846	1106	11	4
15622	3162	11	4
19280	4056	11	4
28878	5722	11	4
31981	13712	11	4
8374	1507	11	4
29148	6322	11	4
7012	1985	11	4
28009	4943	11	4
6046	2003	11	4
14752	2604	11	4
10191	1592	11	4
45606	13448	11	4
9203	1747	11	4
12354	2120	11	4
11995	2538	11	4
41137	11747	11	4
6618	1009	11	4
6904	1693	11	4
29452	3503	11	4
8933	1585	11	4
4244	1323	11	4
10437	1580	11	4
19441	4754	11	4

NUCLEUS AREA	FUORESCENT SIGNAL	PASSAGE NUMBER	EXPERIMENT ID
13235	1718	11	4
7606	1459	11	4
19100	5514	11	4
31201	8033	11	4
22832	11430	11	4
24897	4758	11	4
9031	2489	11	4
15691	3874	11	4
8985	1751	11	4
22527	7729	11	4
13389	3581	11	4
14600	1484	11	4
3834	566	11	4
11621	1597	11	4
9990	2524	11	4
7472	1262	11	4
26564	4534	11	4
25386	9033	11	4
21825	6494	11	4
29754	17920	11	4
28297	7747	11	4
7183	997	11	4
14167	3219	11	4
17276	6156	11	4
11181	2808	11	4
32757	7765	11	4
10897	2357	11	4
13569	3249	11	4
9086	1023	11	4
12934	2757	11	4
19109	7282	11	4
17261	4781	11	4
18417	5127	11	4
25904	4814	11	4
25679	7969	11	4
8251	1235	11	4
35875	12068	11	4
13493	2245	11	4
11601	1999	11	4
21922	7074	11	4
14030	3978	11	4
11251	2295	11	4
15109	2461	11	4
12175	5108	11	4
14364	5009	11	4
12646	3323	11	4
12393	3590	11	4
10249	1743	11	4

NUCLEUS AREA	FUORESCENT SIGNAL	PASSAGE NUMBER	EXPERIMENT ID
24000	5375	11	4
22628	7022	11	4
9708	1971	11	4
10394	3246	11	4
8946	1561	11	4
18279	8039	11	4
5354	1484	10	4
6812	2817	10	4
5450	2020	10	4
6922	2993	10	4
5697	1387	10	4
5392	2886	10	4
11332	3337	10	4
3649	994	10	4
4079	1051	10	4
5550	1249	10	4
5530	1583	10	4
9323	3339	10	4
4938	1219	10	4
7172	1335	10	4
4738	1175	10	4
4617	1221	10	4
6528	1543	10	4
4696	1097	10	4
2412	392	10	4
9755	1555	10	4
2931	395	10	4
9361	1627	10	4
9741	2715	10	4
8861	1708	10	4
7943	1366	10	4
5485	1251	10	4
5045	1012	10	4
6137	1409	10	4
8005	1776	10	4
10950	2717	10	4
5962	1047	10	4
3610	807	10	4
6796	1225	10	4
2029	307	10	4
7857	1283	10	4
6186	935	10	4
5171	1089	10	4
4569	820	10	4
10652	1953	10	4
4141	596	10	4
9840	1917	10	4
10213	3257	10	4

NUCLEUS AREA	FUORESCENT SIGNAL	PASSAGE NUMBER	EXPERIMENT ID
9089	1626	10	4
7937	1186	10	4
5493	1012	10	4
5413	1075	10	4
4597	1993	10	4
5638	2829	10	4
4154	558	10	4
4247	3217	10	4
6739	1299	10	4
3935	2835	10	4
5465	1448	10	4
6835	2550	10	4
8343	1404	10	4
6346	1582	10	4
6980	1332	10	4
3741	566	10	4
7945	1292	10	4
6630	963	10	4
5127	1043	10	4
4156	585	10	4
10148	1403	10	4
4577	996	10	4
13629	4010	10	4
4490	1038	10	4
5563	1052	10	4
5512	1378	10	4
5523	1470	10	4
5324	2736	10	4
6217	3326	10	4
4391	754	10	4
4418	3445	10	4
6861	1463	10	4
4022	2914	10	4
5495	1576	10	4
6887	2684	10	4
8167	1362	10	4
6293	1441	10	4
2086	288	10	4
6889	1214	10	4
4489	1037	10	4
7578	1357	10	4
5650	1516	10	4
2178	611	10	4
7153	1740	10	4
5516	1477	10	4
4384	733	10	4
4003	2896	10	4
4946	1342	10	4

NUCLEUS AREA	FUORESCENT SIGNAL	PASSAGE NUMBER	EXPERIMENT ID
8204	1387	10	4
7489	1285	10	4
4227	519	10	4
6908	1213	10	4
3202	880	10	4
5221	727	10	4
4407	548	10	4
4108	594	10	4
8686	1367	10	4
6452	1606	10	4
6495	1714	10	4
6957	1761	10	4
6584	2077	10	4
7075	2233	10	4
4563	713	10	4
7377	1641	10	4
5102	825	10	4
7517	2363	10	4
4988	814	10	4
4982	1001	10	4
4679	1045	10	4
8021	2223	10	4
6661	1328	10	4
7280	1710	10	4
2921	753	10	4
5735	1221	10	4
6213	1267	10	4
5684	919	10	4
5378	856	10	4
6950	1348	10	4
4178	748	10	4
4354	782	10	4
4638	993	10	4
6935	1318	10	4
7854	2223	10	4
9038	2333	10	4
5711	1164	10	4
7869	1926	10	4
3948	511	10	4
7182	1413	10	4
9714	3168	10	4
7384	2259	10	4
5877	954	10	4
7639	2400	10	4
3209	739	10	4
3398	886	10	4
9506	2241	10	4
2733	343	10	4

NUCLEUS AREA	FUORESCENT SIGNAL	PASSAGE NUMBER	EXPERIMENT ID
8980	2385	10	4
8777	1823	10	4
4554	947	10	4
4804	1052	10	4
8136	3028	10	4
11126	2662	10	4
11158	3261	10	4
11088	1846	10	4
3507	355	10	4
6515	945	10	4
5504	896	10	4
7554	774	10	4
5722	894	10	4
7726	1816	10	4
4671	673	10	4
5236	585	10	4
8846	2950	10	4
8374	2080	10	4
8848	3296	10	4
6799	1049	10	4
6606	1108	10	4
3273	486	10	4
3617	573	10	4
3448	821	10	4
3456	779	10	4
2976	484	10	4
4471	799	10	4
10195	3324	10	4
8307	1613	10	4
6873	2675	10	4
5387	774	10	4
5888	915	10	4
9687	2361	10	4
2362	302	10	4
5129	906	10	4
4127	622	10	4
4735	846	10	4
3931	747	10	4
10776	2829	10	4
4695	702	10	4
5124	605	10	4
4551	554	10	4
4487	490	10	4
5440	822	10	4
6103	1251	10	4
4621	661	10	4
5039	642	10	4
4959	893	10	4

NUCLEUS AREA	FUORESCENT SIGNAL	PASSAGE NUMBER	EXPERIMENT ID
8061	1238	10	4
3570	504	10	4
8401	1524	10	4
5351	891	10	4
4105	808	10	4
4233	786	10	4
7344	1099	10	4
5711	1337	10	4

Table 1. This table contains the data used in this work. Each row represents the data from a single cell in each one of the perfomed experiments. The first column contains the size of the cellular nucleus, estimated with fluorescent probing. The second column contains the raw value of the fluorescence signal returned by the microscope. The third column represent the replicative passage of the observation. The fourth column represent which replication of the experiment was used for the observation.
